# Mass Spectrometry-Based Evaluation of the Bland–Altman Approach: Review, Discussion, and Proposal

**DOI:** 10.3390/molecules28134905

**Published:** 2023-06-21

**Authors:** Dimitrios Tsikas

**Affiliations:** Institute of Toxicology, Core Unit Proteomics, Hannover Medical School, 30623 Hannover, Germany; tsikas.dimitros@mh-hannover.de

**Keywords:** agreement, biomarkers, Bland and Altman approach, comparison, linear regression analysis, mass spectrometry, Oldham, Eksborg, tandem mass spectrometry, validation

## Abstract

Reliable quantification in biological systems of endogenous low- and high-molecular substances, drugs and their metabolites, is of particular importance in diagnosis and therapy, and in basic and clinical research. The analytical characteristics of analytical approaches have many differences, including in core features such as accuracy, precision, specificity, and limits of detection (LOD) and quantitation (LOQ). Several different mathematic approaches were developed and used for the comparison of two analytical methods applied to the same chemical compound in the same biological sample. Generally, comparisons of results obtained by two analytical methods yields different quantitative results. Yet, which mathematical approach gives the most reliable results? Which mathematical approach is best suited to demonstrate agreement between the methods, or the superiority of an analytical method A over analytical method B? The simplest and most frequently used method of comparison is the linear regression analysis of data observed by method A (*y*) and the data observed by method B (*x*): *y* = α + β*x*. In 1986, Bland and Altman indicated that linear regression analysis, notably the use of the correlation coefficient, is inappropriate for method-comparison. Instead, Bland and Altman have suggested an alternative approach, which is generally known as the Bland–Altman approach. Originally, this method of comparison was applied in medicine, for instance, to measure blood pressure by two devices. The Bland–Altman approach was rapidly adapted in analytical chemistry and in clinical chemistry. To date, the approach suggested by Bland–Altman approach is one of the most widely used mathematical approaches for method-comparison. With about 37,000 citations, the original paper published in the journal *The Lancet* in 1986 is among the most frequently cited scientific papers in this area to date. Nevertheless, the Bland–Altman approach has not been really set on a quantitative basis. No criteria have been proposed thus far, in which the Bland–Altman approach can form the basis on which analytical agreement or the better analytical method can be demonstrated. In this article, the Bland–Altman approach is re-valuated from a quantitative bioanalytical perspective, and an attempt is made to propose acceptance criteria. For this purpose, different analytical methods were compared with *Gold Standard* analytical methods based on mass spectrometry (MS) and tandem mass spectrometry (MS/MS), i.e., GC-MS, GC-MS/MS, LC-MS and LC-MS/MS. Other chromatographic and non-chromatographic methods were also considered. The results for several different endogenous substances, including nitrate, anandamide, homoarginine, creatinine and malondialdehyde in human plasma, serum and urine are discussed. In addition to the Bland–Altman approach, linear regression analysis and the Oldham–Eksborg method-comparison approaches were used and compared. Special emphasis was given to the relation of difference and mean in the Bland–Altman approach. Currently available guidelines for method validation were also considered. Acceptance criteria for method agreement were proposed, including the slope and correlation coefficient in linear regression, and the coefficient of variation for the percentage difference in the Bland–Altman and Oldham–Eksborg approaches.

## 1. Introduction

Most likely, nobody knows how many low- and high-molecular-mass chemical compounds are present in biological samples such as blood and urine. Yet, their number is assumed to be very high and to increase with time due to the discovery of natural and the introduction into the environment of new synthetic compounds including drugs. The core mission of *Analytical Chemistry* is both to identify the structure these compounds and to determine their concentration as accurately as possible. Over the years, numerous analytical methods were reported for the quantitative determination of virtually all classes of chemical compounds. Scientific competition, curiosity and striving, often paired with the discovery of novel technologies and improvements in available methodologies, have resulted in, and consistently result in, the development of various analytical methods in part for the same analyte, yet with different analytical performances. The performance of analytical methods can be characterized with a certain degree of objectivity, especially when defined criteria are applied. Generally, an improvement in a current analytical method for a certain analyte is an acceptable justification for the publication of the improved analytical method in a scientific journal, despite its lacking true analytical novelty.

### 1.1. Method-Comparison Approaches

Method-comparison approaches were proposed, interpreted, discussed, criticized, and improved by several groups [1,2,3,4,5,6,7,8,9,10,11,12,13,14,15,16,17,18,19,20,21,22,23,24,25,26,27,28,29,30,31,32,33,34,35,36] (in part cited in chronological order). Linear regression analysis (see Formula (1)) of results obtained by two different methods for the same measure, e.g., for an analyte in a biological system, is reportedly the oldest approach of method-comparison. In 1986, Bland and Altman published in *The Lancet* their legendary paper entitled *Statistical methods for assessing agreement between two methods of clinical measurement* [10]. This paper is one of the most frequently cited articles in Life Sciences (Figure S1). Bland and Altman [10] have noted that linear regression analysis, notably the use of the correlation coefficient *r*, is inappropriate for method-comparison, and they have suggested an alternative approach. Despite the availability of approaches for stronger analytical power, including the Bland–Altman method, linear regression analysis is still, without doubt, the most frequently and routinely used approach in the field of analytical chemistry and in other areas, until this day. Interestingly, the Bland–Altman method seems to be more widespread in the field of clinical chemistry. The consequences of the use of inappropriate or unsatisfactory approaches for method-comparison, such as the sole use of any value of the correlation coefficient *r*, as long it is associated with a statistically significant *p* value, i.e., *p* ≤ 0.05, may be grave, as is demonstrated in the present study.

*τ*_1_*_j_* = α + β × *τ*_2*j*_ (1)

whereas *τ*_1*j*_ and *τ*_2*j*_ are the values measured by method 1 and method 2 (*j* = 1, 2, … *n*−1, *n*; *n* = total number of the analyzed samples), respectively; α and β are values of the *y*-axis intercept and the slope of the straight line, respectively.

The Bland–Altman (BA) method [10] is a rather graphical approach which is still widely used but is less frequently and not routinely applied in analytical chemistry. The Bland–Altman approach examines the relationship between the difference (δ_BA_ or simply δ) of the values obtained by two methods (see Formula (2)) and the mean (μ_BA_ or simply μ) of the methods (see Formula (3)). Usually, in this approach, δ_BA_ is plotted versus the μ_BA_ of the methods.

δ_BA_ = *τ*_1*j*_ − *τ*_2*j*_ (2)

µ_BA_ = 1/2 × (*τ*_1*j*_ + *τ*_2*j*_) (3)

Even if the Bland–Altman approach is steadily used in analytical chemistry, this method-comparison is applied incorrectly, most likely due to the lack of acceptance criteria. Thus, most of the measurements may be within a 95% confidence interval, e.g., the ±1.96 × standard deviation, despite lacking analytically relevant comparability. This is because the 95% confidence interval becomes wider the larger the difference between the methods is (see below). It should be emphasized that Bland and Altman suggested their approach for quite comparable methods [10]. In this respect, both linear regression analysis and the Bland–Altman approach are used arbitrarily and do not provide reliable information about a potentially existing comparability and the extent of agreement.

Oldham [2], and later Eksborg [8], have suggested independently of each other an alternative approach which is based on using the ratio (Λ_OE_ or simply Λ; see Formulas (4a) and (4b)) of the measured values by two methods versus the mean of the values or versus the values *τ*_2_ of method 2 (chosen as the reference method; Formula (4a)) or versus their average (Formula (4b)). In the present work, this method is referred to as OE. Interestingly, and in the opinion of this author, surprisingly, this approach did not find appreciable applications in method-comparison studies until the present day.

Λ_OE_ = *τ*_1*j*_:*τ*_2*j*_ (4a)

Λ_OE_ = *τ*_1*j*_:0.5 × (*τ*_1*j*_ + *τ*_2*j*_) (4b)

### 1.2. Basic Principles of Mass Spectrometry and Tandem Mass Spectrometry

Analytical methods involving the use of mass spectrometers, such as gas chromatography-mass spectrometry (GC-MS) and liquid chromatography-mass spectrometry (LC-MS) apparatus, are based on the separation of inorganic and organic ions produced in the ion-source of the instruments due to their mass-to-charge (*m*/*z*) ratio (Scheme S1). This ability provides mass spectrometry (MS)-based approaches with inherent specificity and distinguishes them from other analytical techniques, which are based on the utilization of far less characteristic physicochemical properties such as light absorption, fluorescence, or conductivity. The separation of substances by their *m*/*z* values enables the use of stable-isotope-labelled analogues as internal standards (IS) in MS-based methods. This is a unique feature of MS technology and lends MS-based methods high accuracy in quantitative analysis. A quantum jump in specificity and accuracy is represented by tandem mass spectrometry (MS/MS), for instance, as realized in GC-MS/MS and LC-MS/MS instruments. Not without reason, MS/MS-based methods are regarded as the *Reference Methods*, the *Gold Standard*, in the area of analytical chemistry, in basic and clinical research, including clinical chemistry (see for instance Refs. [37,38,39,40]).

In biological samples, such as plasma and urine samples, there are myriads of substances that belong to distinctly different classes. In MS-based methods, sample treatment procedures, such as protein precipitation, proper extraction and/or derivatization prior to analysis, generally lead to a considerable reduction in the number of the analytes finally injected into the MS instrument. The number of analytes that may interfere with the analysis of a certain substance may be further reduced by gas chromatographic or liquid chromatographic separation prior to MS separation. Despite a strong reduction in the number of potentially interfering analytes by such steps, the ionization process of co-eluting substances may generate isobaric ions, i.e., structurally different ions which have, however, the same *m*/*z* value. This particular situation is illustrated in Scheme S1 for mass spectrometers based on quadrupole (Q) technology.

Commonly, quantification by GC-MS and LC-MS instruments (and by GC-MS/MS and LC-MS/MS instruments operated in the SSQ configuration) is performed in the selected-ion monitoring (SIM) mode, as shown in Scheme S2A. In general, two ions produced in the ion source are selected: one ion for the target analyte A_T_ and one ion for the corresponding ion of the stable-isotope-labelled analogue A_IS_, which serves as the IS for the analyte A_T_. Quantification by GC-MS/MS and LC-MS/MS instruments is usually carried out in the selected-reaction monitoring (SRM) mode, as illustrated in Scheme S2B. For example, from the ions produced in the ion source of a GC-MS/MS instrument, the first quadrupole Q1 alternately separates the ion with *m*/*z* A_T_ for the target analyte and the corresponding ion *m*/*z* A_IS_ for the externally added IS. These precursor ions are fragmented in the collision chamber (second quadrupole) Q2, and the third quadrupole Q3 alternately selects, in general, each one specific product ion (*p*); for instance, it “filters” *m/z* P_T_ for the target analyte and *m*/*z* P_IS_ for the IS (Scheme S2B). In the SRM mode, Q3 can also pass product ions of the same *m*/*z* value (i.e., *m*/*z* P_T_ = *m*/*z* P_IS_) which are, however, produced from different precursor ions and can therefore be completely discriminated.

A more detailed description of the instrumentation and principles of operation techniques, including ionization techniques, with SSQ and TSQ mass spectrometers and other types of mass spectrometers can be referred to the literature (e.g., Refs. [41,42,43,44,45]). A history of European mass spectrometry is found in Ref. [46]. In the context of quantitative analytical chemistry, it should be emphasized that mass spectrometry is not, per se, a magic bullet, and it does not always guarantee valid data [47]. Yet, it is currently the best available technology in analytical chemistry. It must be used with validated methods, and all findings need to be critically evaluated [48].

### 1.3. Problem and Aim of the Study

Because of the lack of guide numbers for the correlation coefficient *r*, the *y*-axis intercept α and the slope β of the regression equation, the results of linear regression analysis for method-comparison are used rather arbitrarily [10,49]. In particular, the lack of a definition of the acceptance criteria for the correlation coefficient *r* seduces us into misusing regression analysis, for instance, into suggesting an agreement in doubtful cases or even in missing analytical agreements. Thus, even if the value for the correlation coefficient *r* is, for instance, only 0.8, a *p* value below 0.05 is commonly considered satisfactory to claim correlation between the methods tested, irrespective of the *y*-axis intercept and slope values of regression equations.

In principle, these considerations equally apply to both the Bland–Altman method and the Oldham–Eksborg approach. Actually, these two methods lack a definition of the acceptance criteria for comparability and the validity of the analytical methods being compared. The Bland–Altman approach is useful for comparisons of methods with comparable performances, as originally stated by Bland and Altman in their original work [10]. In cases of considerable disagreement between the methods being compared, the Bland–Altman approach would penalize the method with the better analytical performance, e.g., method 2, in favour of the method with the putatively lower-quality analytical method, e.g., method 1. For example, this could be the case when comparing GC-MS/MS or LC-MS/MS methods with GC-FID or HPLC-UV methods. Application of the Bland–Altman approach to two methods being less comparable would result into a too-large confidence interval. Thus far, no additional established quantitative parameters of this approach have been proposed to value and report the extent of the agreements between methods. Regrettably, and in analogy to regression analysis, the Bland–Altman approach is interpreted incorrectly by many investigators, presumably because of the lack of acceptance criteria. Commonly, the application of this approach to method-comparison is solely restricted to showing the graph and the confidence interval. Eventually, the Oldham–Eksborg approach finds generally very few applications in method-comparisons despite the considerable potential of this method.

As will be shown in this work, the approaches of linear regression analysis, Bland–Altman, and Oldham–Eksborg are linked together. Therefore, one possibility to overcome the flaws of the individual approaches could be the deviation and use of a proper combination of these methods. However, even if this would be profitable, it may not allow us to solve the main, common, and principal problem of method-comparison: the renunciation of the superiority of one method over the other method, or the arbitrariness of defining one of the methods being compared as the *absolute* reference method, the *Gold Standard*. We could extricate ourselves from this dilemma if we accepted that thoroughly validated and proven analytical methods based on the tandem mass spectrometry methodology, such as GC-MS/MS and LC-MS/MS methods, are best-qualified to represent the reference methods [48]. The superiority of tandem mass spectrometry technology over other putatively less reliable analytical techniques is reasonably indisputable in the literature. The examples presented below are supportive of the analytical superiority of the MS/MS methodology over other analytical methodologies.

The aim of the present study was to investigate whether or not defining validated and proven analytical MS/MS-based methods as reference methods may help solve problems associated with method-comparison and may even help to define acceptance criteria for linear regression analysis, the Bland–Altman, and the Oldham–Eksborg approaches. Most currently available guidelines proposed by international associations and analytically oriented journals address exogenous drugs as analytes [50,51,52,53,54,55,56] rather than endogenous substances which have special requirements beyond method validation [57]. The present work focuses on the quantitative analysis of endogenous substances in biological samples, which represents a formidable analytical challenge.

## 2. Methods

### 2.1. Re-Evaluation of Published Analytical Data

#### Proceeding

**Scheme 1:**
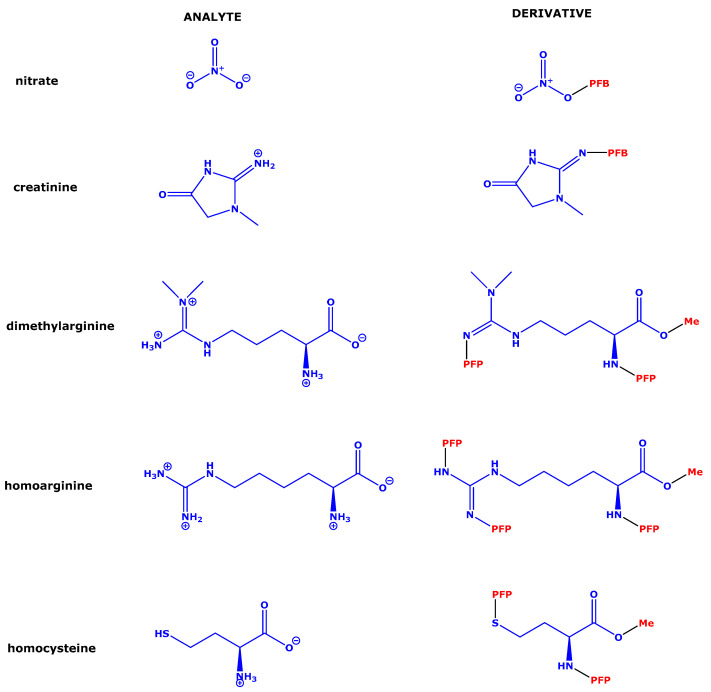
Chemical structures of the native analytes (**left**) and their chemical derivatives (**right**) discussed in the present work. Nitrate, nitrite, malondialdehyde, and creatinine are derivatized with pentafluorobenzyl bromide in aqueous acetone (e.g., 60 min at 50 °C). Asymmetric dimethylarginine (ADMA), homoarginine and homocysteine are first methylated in 2 M HCl in methanol (e.g., 30 min at 80 °C), and then by pentafluoropropionic anhydride in ethyl acetate (e.g., 60 min at 65 °C). Me, methyl; PFB, Pentafluorobenzyl; PFP, pentafluoropropionyl.

Selected studies published by the author’s group and by other investigator groups were examined by three method-comparison approaches: (1) linear regression analysis; (2) the Bland–Altman (BA) method; and (3) the Oldham–Eksborg (OE) approach. The selected studies reported results which allow for a satisfactory re-evaluation. The data reported in the Figures and Tables of this article were reconstituted and re-evaluated by the author to the best of his ability. For simplicity, values in Tables are reported without their respective units. Statistical data from the author’s group were generated using GraphPad Prism Version 7 for Windows (GraphPad Software, San Diego, CA, USA). Chemical structures were drawn using ChemDraw 15.0 Professional (PerkinElmer, Germany). The structures of some analytes discussed in the present work are illustrated in Scheme 1. Where applicable, data analysis is reported in the following sections in more detail.

**Figure 1:**
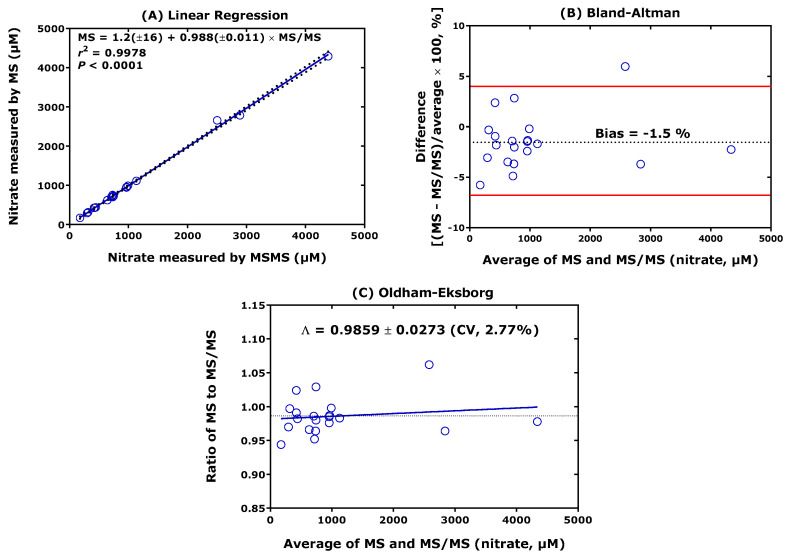
Measurement of nitrate in human urine by GC-MS (i.e., MS, method (1) and GC-MS/MS (i.e., MS/MS, method (2) and their comparison by: (**A**) linear regression; (**B**) Bland–Altman; and (**C**) Oldham–Eksborg. This Figure was constructed by using the data of Table 1 of the article [58]. Samples were analyzed on the instrument TSQ 7000 first by GC-MS in the SIM mode and subsequently by GC-MS/MS in the SRM mode. The closed points in (**A**) indicate the 95% confidence bands. Horizontal solid lines in (**B**) indicate the 95% limits of agreement (±1.96 × SD).

**Figure 2:**
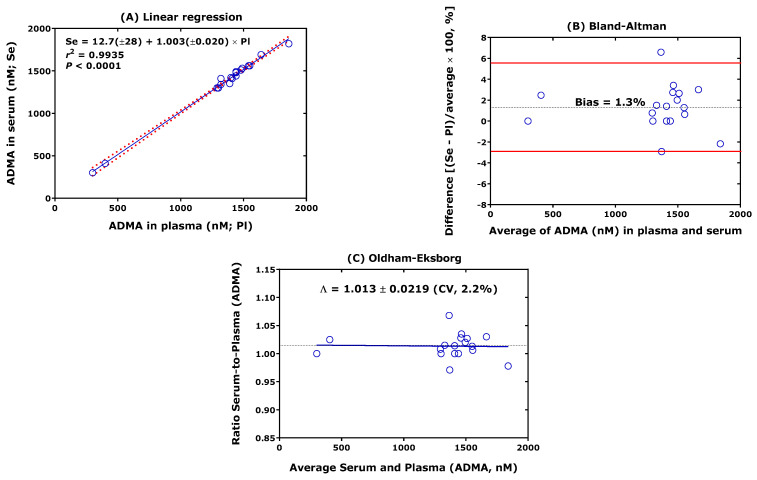
Comparison of measurements of asymmetric dimethylarginine (ADMA) in human plasma (method 1) and serum (method 2) of one patient with acute renal failure before, during and after extended haemodialysis for 8 h: (**A**) linear regression analysis; (**B**) Bland–Altman; and (**C**) Oldham–Eksborg. This Figure was constructed by using the data of Table 1 of a previous article [62]. Samples were analyzed for ADMA on the instrument TSQ 7000 by GC-MS/MS in the SRM mode as reported elsewhere [63]. Horizontal solid lines in (**B**) indicate the 95% limits of agreement.

**Figure 3:**
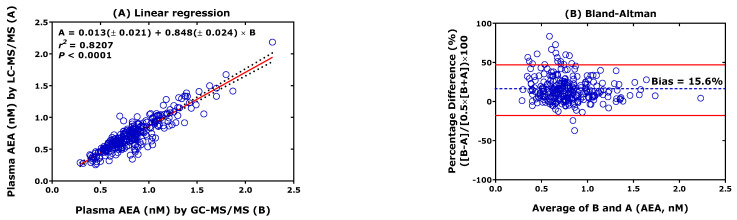

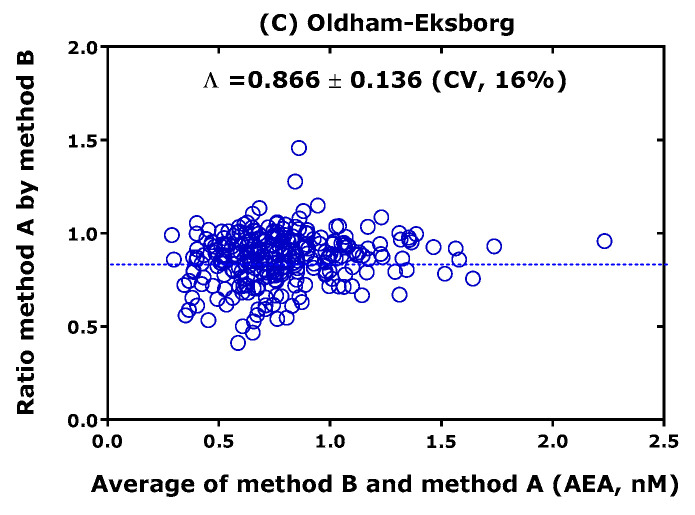
Comparison of measurements of anandamide (AEA) in 277 human plasma samples by LC-MS/MS (method 1) and by GC-MS/MS (method 2): (**A**) linear regression analysis; (**B**) Bland–Altman; and (**C**) Oldham–Eksborg. Samples were analyzed for AEA on the instrument TSQ 7000 by GC-MS/MS [68] and by Xevo LC-MS/MS [69] as reported in these references. Horizontal solid lines in (**B**) indicate the 95% limits of agreement.

**Figure 4:**
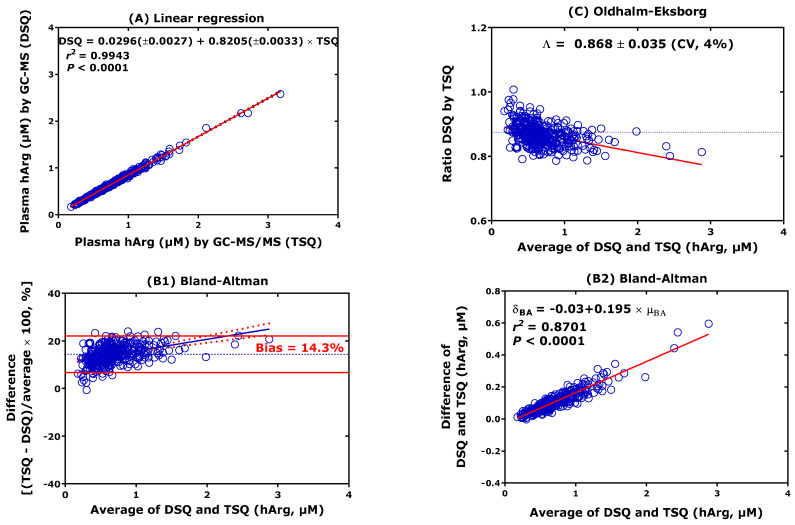
Comparison of measurements of homoarginine (hArg) in plasma by GC-MS (method 1) and by GC-MS/MS (method 2) in 369 plasma samples of pregnant women: (**A**) linear regression analysis; (**B1,B2**) Bland–Altman; and (**C**) Oldham–Eksborg. Within (**B1,B2**): linear regression analysis between percentage difference and average. This Figure was constructed by using the data published in a previous article [70]. Note that two different apparatus were used: Samples were analyzed for hArg on the instrument TSQ 7000 by GC-MS/MS in the SRM mode and on the instrument DSQ in the SIM mode as reported elsewhere [70]. Horizontal solid lines in (**B1**) indicate the 95% limits of agreement. Note that the difference between the methods is used in percentages ((**B1**), %) and in absolute concentrations ((**B2**), µM).

**Figure 5:**
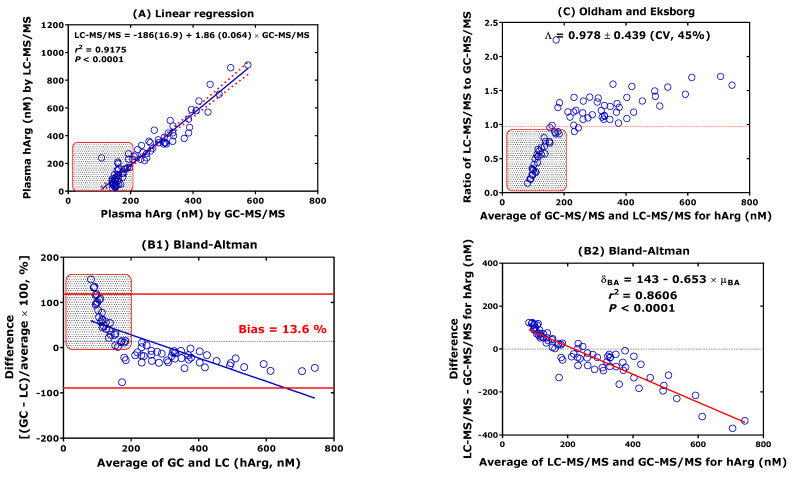
Comparison of measurements of homoarginine (hArg) in mouse plasma by GC-MS/MS (method A) and by LC-MS/MS (method B) in 79 plasma samples: (**A**) linear regression analysis; (**B1,B2**) Bland–Altman; and (**C**) Oldham–Eksborg. Within (**B1,B2**): linear regression analysis between percentage difference and average. This Figure was constructed by using the data published in a previous article [71]. Note that two different apparatus were used: Samples were analyzed for hArg on the instrument TSQ 7000 by GC-MS/MS in the SRM mode and on the instrument Varian 1200 L Triple Quadrupole MS in the SRM mode as reported elsewhere [71]. Horizontal solid lines in (**B1,B2**) indicate the 95% limits of agreement. Shaded insets indicate ranges of maximum disagreement.

**Figure 6:**
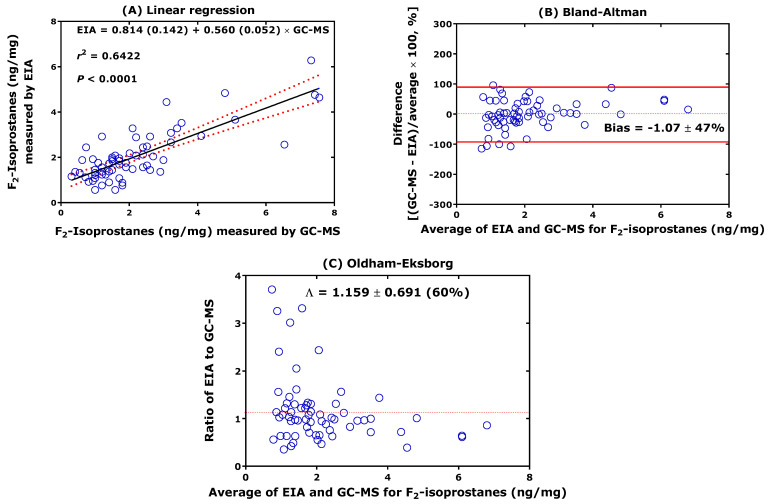
This Figure was constructed by using the data of Figure 1 of the article by Devaraj et al. [73]: comparison of a commercially available EIA method with a GC-MS method [72] for F_2_-isoprostanes in urine; data are reported as ng F_2_-isoprostanes per mg creatinine in this article: (**A**) linear regression analysis; (**B**) Bland–Altman; and (**C**) Oldham–Eksborg. Horizontal dotted lines indicate ±2 SD range. See Refs. [72,73,74,79,88,89,90,91] regarding analysis of F_2_-isoprostanes.

**Figure 7:**
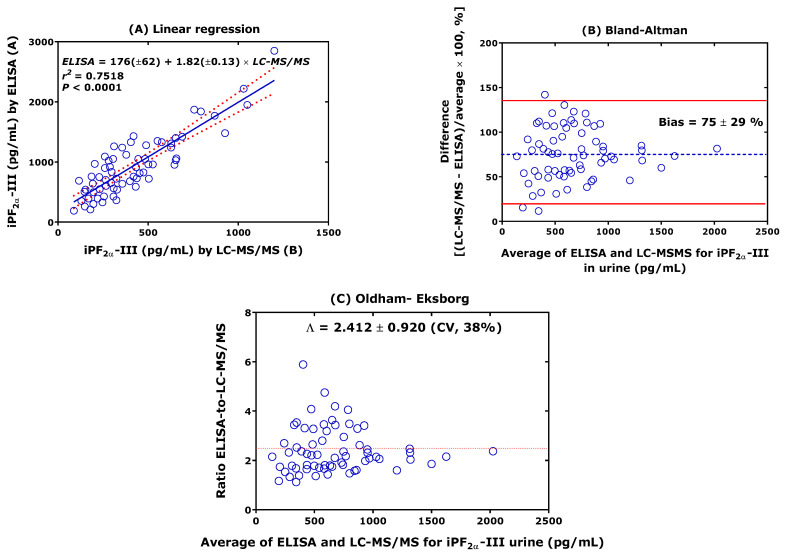
This Figure was constructed by using the data of Figure 5 of the article by Yan et al. [74]; due to considerable overlap of data points not all data points of the original Figure 5 from Ref. [74] could be used in the present work. Comparison of a commercially available ELISA method with a LC-MS/MS method for IPF_2α_-III (8-*iso*-PGF_2α_) in urine by: (**A**) linear regression analysis, (**B**) Bland–Altman; and (**C**) Oldham–Eksborg. Horizontal dotted lines indicate ±2 × SD range in (**B**). See Refs. [72,73,74,79,88,89,90,91] regarding analysis of F_2_-isoprostanes.

**Figure 8:**
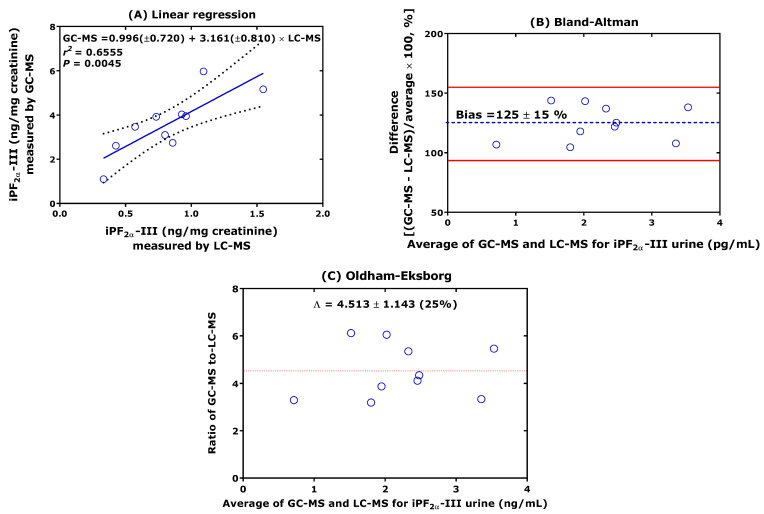
This Figure was constructed by using the data of Figure 4 of the article by Sircar and Subbaiah [90]. Comparison of an LC-MS method with a GC-MS method for IPF_2α_-III (8-*iso*-PGF_2α_) in urine by (**A**) linear regression analysis, (**B**) Bland–Altman, and (**C**) Oldham–Eksborg. Horizontal dotted lines indicate ± 2 SD range in (**B**). See Refs. [72,73,74,79,88,89,90,91] regarding analysis of F_2_-isoprostanes.

**Figure 9:**
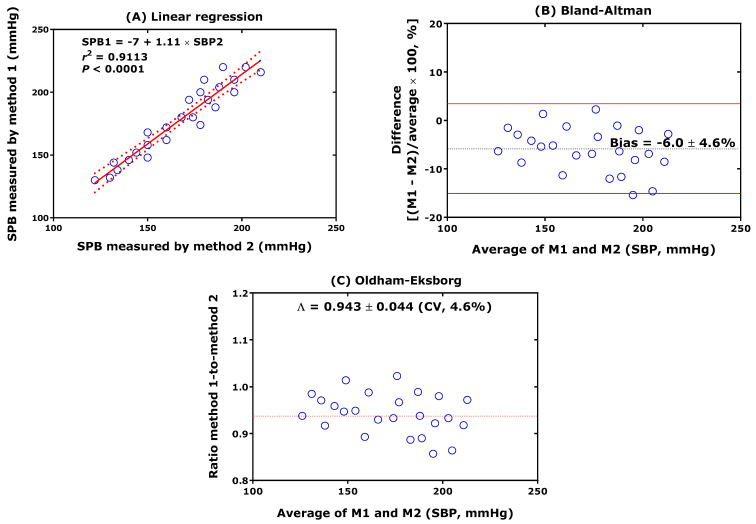
This Figure was constructed by using the data of Table 1 of the article by Ludbrook [75] which had been lent out from Ref. [76]. Comparison of measuring systolic blood pressure (SBP) by method 1 (M1) and method 2 (M2) in 25 patients with essential hypertension; method 2 was chosen arbitrarily as the reference method by the author of the present article. (**A**) Linear regression analysis; (**B**) Bland–Altman method. (**C**) Oldham–Eksborg method.

**Figure 10:**
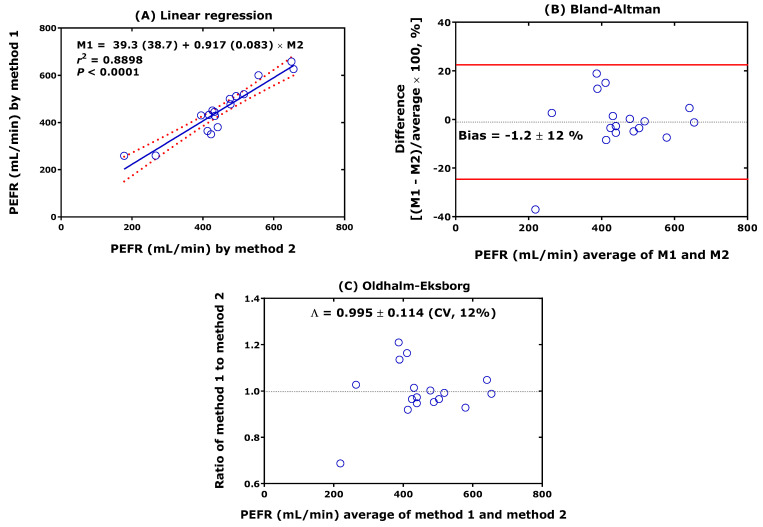
This Figure was constructed by using the data of the Table of the article by Bland and Altman [10]. Comparison of measuring peak expiratory flow rate (PEFR) by method 1 (M1) and method 2 (M2) in 17 subjects; method 2 was chosen arbitrarily as the reference method by the author of the present article. (**A**) Linear regression analysis; (**B**) Bland–Altman method. (**C**) Oldham–Eksborg method.

**Figure 11:**
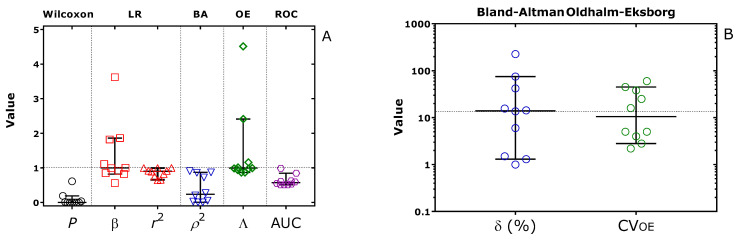
Presentation of values for selected statistical parameters of Table 1 obtained from the Wilcoxon or paired *t* test (*p*), the linear regression analysis (β and *r*^2^), the coefficient of correlation *ρ*^2^ from the linear regression analysis of the Bland–Altman difference δ vs. the average concentration of the analyte, the Oldham–Eksborg ratio Λ, the AUC value from the ROC analysis (**A**); and of the percentage difference of the Bland–Altman test δ (%), and the coefficient of variation of the Oldham–Eksborg ratio CV_OE_ (**B**). Data from 10 examples were used. Because of the greatly differing size of the values, the data are presented in two panels. Note the decadic logarithmic scale on the *y* axis in panel B. Data are shown as median with 95% confidence interval. LR, linear regression. See Table 1.

Standard analytical parameters included in the present work are: (1) *y*-axis intercept α, slope β, and goodness of fit (*r*^2^) from linear regression analysis; (2) the mean of the difference δ_BA_, the average µ_BA_ and the bias values from the BA approach; and (3) the OE ratio Λ_OE_. In addition, further statistically relevant parameters, notably the relative standard deviation (RSD) or coefficient of variation (CV) of the absolute difference δ and the percentage difference δ(%), and of the ratio Λ_OE_ were included. In the BA approach, linear regression analysis between δ or δ(%) versus the average was performed and the goodness of fit (*ρ*^2^) was reported. It is assumed that these measures allow for evaluations of agreement between two methods more effectively and on a quantitative basis as compared to the rather qualitative information provided by the individual approaches. In addition, the receiver operating characteristic (ROC) approach was used and the area under the curve (AUC) values were considered to evaluate agreement/disagreement between two compared methods. The complete set of the results from the meta-analyzed studies is presented in Figures 1–11 and summarized in Table 1.

### 2.2. Measurement of Nitrate in Human Urine-Comparison of GC-MS with GC-MS/MS

Nitrate (Scheme 1) is the major circulating and urinary metabolite of nitric oxide (NO) [81]. Nitrate in urine is a suitable measure of whole-body NO synthesis. Figure 1 shows the results from the re-evaluation of data previously reported by our group (Table 1 of Ref. [58]) regarding validation by GC-MS/MS of a GC-MS method for the quantitative analysis of nitrate in human urine. In the urine samples analyzed, the nitrate concentration ranged between about 100 µM and 4000 µM. The nitrate concentration was measured to be (mean ± SD) 1048 ± 1024 µM (CV, 98%) by GC-MS (method 1) and 1059 ± 1035 µM (CV, 98%) by GC-MS/MS (method 2). The values differed between the methods (*p* = 0.014; two-tailed Wilcoxon test).

Linear regression analysis between the data obtained by GC-MS and those by GC-MS/MS resulted in a regression equation with a very low *y*-axis intercept value α = 1.2, a slope value β = 0.988 close to unity, and a very high correlation coefficient (*r*^2^ = 0.9978). These observations suggest a very tight agreement between the GC-MS (method 1) and the GC-MS/MS (reference method 2) (Figure 1A).

The Bland–Altman approach revealed a very low difference between the two methods δ_BA_ = −12 ± 50 µM according to a percentage difference δ (%) of −1.5 ± 2.7% (mean ± SD), which is only a very small portion of the mean concentration of nitrate measured in the whole concentration range (Figure 1B). Neither the difference (*ρ*^2^ = 0.05) nor the percentage difference (*ρ*^2^ = 0.02) correlated with the average concentration. Thus, the findings argue for a close agreement between the GC-MS and GC-MS/MS methods for nitrate in human urine.

The approach according to Oldham and Eksborg gave a concentration-independent ratio Λ_OE_ = 0.986 ± 0.027 which is very close to the unity and has a low CV value of only 2.8% (Figure 1C). In the Bland–Altman approach, the ratio of the two methods can also be plotted against the average of the two methods. It provided a value of 0.9858 ± 0.027 which is identical to the ratio Λ_OE_. The third approach used to compare the GC-MS method with the GC-MS/MS method for urinary nitrate strongly indicates that the GC-MS method is as suitable as the GC-MS/MS method for the accurate quantitative determination of nitrate in human urine.

The ROC approach on these data resulted in the AUC value of 0.531 ± 0.093 and a *p* value of *p* = 0.735. This data can be interpreted as having a high agreement between the two methods.

### 2.3. Measurement of Asymmetric Dimethylarginine (ADMA) in Human Plasma and Serum

Asymmetric dimethylarginine (ADMA) is an endogenous inhibitor of NO synthase (NOS), which catalyzes the conversion of L-arginine to NO and is a cardiovascular risk factor [81]. ADMA circulates in blood and is excreted in the urine. Several methods were developed for the measurement of ADMA mainly in plasma and serum [63,64,65,66,67,82,83,84]. The concentration of ADMA in serum and heparinized plasma of humans was reported by many groups using HPLC, GC-MS, GC-MS/MS and LC-MS/MS methods and found not to differ significantly, with deviations being in the order of 1% [82,83,84].

We have utilized this feature of ADMA and quantitated ADMA by GC-MS/MS in heparinized plasma and serum samples generated from blood samples of a patient suffering from end-stage kidney disease before, during, and after extended haemodialysis for 8 h [62]. The ADMA concentration (mean ± SD) was measured to be 1351 ± 386 nM (CV, 29%) in serum (method 2) and 1334 ± 383 nM (CV, 29%) in plasma (method 1). The values differed between the methods (*p* = 0.034; two-tailed Wilcoxon test). The results of the methods-comparison are shown in Figure 2.

Linear regression analysis between the data measured in serum (method 2) and those in plasma (method 1) resulted in a regression equation with a very low *y*-axis intercept value α = 12.7, a slope value β = 1.003 very close to the unity, and a very high correlation coefficient (*r*^2^ = 0.9935). These observations suggest a very high agreement between the serum and the plasma ADMA levels (Figure 2A).

The Bland–Altman approach revealed a very low difference between the two methods (17.2 ± 1.2 nM) according to a percentage difference of 1.3 ± 2.1% (bias), which is only a very small portion of the mean concentration of ADMA measured in the whole concentration range (Figure 2B). Neither the difference (*ρ*^2^ = 0.007) nor the percentage difference (*ρ*^2^ = 0.001) correlated with the average concentration. These findings argue for a close agreement between the plasma and serum “methods” for ADMA.

The approach according to Oldham and Eksborg gave Λ_OE_ = 1.013 ± 0.0219 which is very close to the unity and has a CV value of only 2.2% (Figure 2C). Thus, the third approach that was used to compare the serum and plasma levels of ADMA strongly indicates that ADMA can be measured equally accurately in human serum and plasma samples by GC-MS/MS.

The ROC approach on these data resulted in the AUC value of 0.549 ± 0.097 (*p* = 0.613), suggesting no difference, i.e., a high extent of agreement between the two methods.

### 2.4. Measurement of Anandamide in Human Plasma by GC-MS/MS and LC-MS/MS

Anandamide (AEA) is an endogenous cannabinoid, an endocannabinoid, and is mainly measured in human plasma and serum at concentrations in the upper pM-to-the lower nM range [68,69,85,86]. For the measurement of AEA in human plasma, we developed GC-MS/MS [68] and LC-MS/MS [69] methods, which utilize stable-isotope-labelled AEA as the internal standard. In the GC-MS/MS method, AEA is derivatized, while in the LC-MS/MS method AEA is analyzed without derivatization. We compared these methods by parallel measurements of AEA in the plasma of healthy humans. In this comparison, we considered the GC-MS/MS method as the reference method.

The AEA concentration (mean ± SD) was measured to be 0.844 ± 0.289 nM (CV, 34%) by GC-MS/MS (method 2) and 0.729 ± 0.270 nM (CV, 37%) by LC-MS/MS (method 1). The values differed between the methods (*p* < 0.0001; two-tailed Wilcoxon test). The results of the methods-comparison are shown in Figure 3.

The linear regression analysis between the AEA concentrations measured by LC-MS/MS and those measured by GC-MS/MS resulted in a regression equation with a *y*-axis intercept value α = 0.013, a slope value β = 0.848, and a relatively small correlation coefficient (*r*^2^ = 0.8207). These observations suggest a weak agreement between LC-MS/MS and GC-MS/MS (Figure 3A).

The Bland–Altman approach revealed a low but considerably varying difference between the two methods (0.116 ± 0.123 nM; CV, 106%) according to a percentage difference of 15.6% (bias) (Figure 3B). Neither the difference (*ρ*^2^ = 0.024) nor the percentage difference (*ρ*^2^ = 0.024) correlated with the average concentration. These findings argue for a weak agreement between the LC-MS/MS and GC-MS/MS methods regarding AEA measurement in human plasma samples.

The approach according to Oldham and Eksborg resulted in the ratio Λ_OE_ = 0.866 ± 0.136 which is not close to the unity and has a moderate variation (CV, 16%) (Figure 3C). Thus, the third OE-approach confirms the results of the linear regression and Bland–Altman method.

The ROC approach yielded an AUC value of 0.626 ± 0.024 and a *p* value < 0.0001, suggesting some extent of agreement between the two methods.

In summary, GC-MS/MS and LC-MS/MS are suitable for the measurement of AEA in human plasma but yield considerably different results.

### 2.5. Measurement of Homoarginine in Human Plasma by GC-MS and GC-MS/MS-Real Data and a Simulation

L-Homoarginine (hArg) is a non-proteinogenic amino acid. Low plasma and urinary hArg concentrations are considered to be risk markers for cardiovascular and renal diseases [87]. For the quantitative determination of hArg in human plasma, serum and urine samples, we have developed GC-MS and GC-MS/MS methods using L-[^2^H_3_]homoarginine (d_3_-hArg) as the internal standard [86]. In healthy humans, plasma, serum, and urine concentrations of hArg are on the order of 2–3 µM [87].

The hArg concentration (mean ± SD) was measured to be 0.747 ± 0.367 µM (CV, 49%) by GC-MS/MS (method 2) and 0.643 ± 0.302 µM (CV, 47%) by GC-MS (method 1). The values differed between the methods (*p* < 0.0001; two-tailed Wilcoxon test). The results of the methods-comparison are shown in Figure 4.

Linear regression analysis between the hArg concentrations measured by GC-MS (method 1, DSQ) and those measured by GC-MS/MS (method 2, TSQ) resulted in the regression equation with a *y*-axis intercept value α = 0.030, a slope value β = 0.821, and a correlation coefficient (*r*^2^ = 0.9943). These observations suggest a close correlation between GC-MS and GC-MS/MS, with the GC-MS method providing constantly lower hArg concentrations than TSQ (Figure 4A).

The Bland–Altman approach revealed a relatively low difference between the two methods (0.105 ± 0.070 µM) according to a percentage difference of 14.3 ± 27% (bias) (Figure 4B1). The percentage difference (*y*) correlated very weakly (*ρ*^2^ = 0.1739, *p* < 0.0001) with the average concentration of hArg (*x*): *y* = 10.9 + 4.9 × *x*. The difference correlated more strongly (*ρ*^2^ = 0.8701, *p* < 0.0001) with the average concentration of hArg: *y* = −0.03 + 0.195 × *x* (Figure 4B2). These findings argue for a considerable agreement between the GC-MS and GC-MS/MS methods regarding hArg measurement in human plasma samples, with the GC-MS method resulting in constantly lower hArg concentrations. 

The approach according to Oldham and Eksborg resulted in the ratio Λ_OE_ = 0.868 ± 0.035 (CV, 4%) which is not close to the unity (Figure 4C). Linear regression between the Λ_OE_ ratio (*y*) and the average (*x*) resulted in the regression equation *y* = 0.897 − 0.043 × *x* (*r*^2^ = 0.173, *p* < 0.0001), indicating weak concentration-dependency.

The ROC approach yielded and AUC value of 0.596 ± 0.021 and a *p* value < 0.0001, suggesting some extent of agreement between the two methods.

The hArg concentrations measured by DSQ (i.e., by GC-MS) were changed by multiplication to reach higher (DSQ × 1.2) and lower (DSQ × 0.8, DSQ × 0.6) concentrations. All methods of comparison were used to compared unchanged and changed hArg concentrations with those measured by TSQ (i.e., by GC-MS/MS). The results of these analyses are summarized in Table 2.

The best agreement between TSQ and DSQ were observed between plasma hArg concentrations measured by TSQ and by DSQ × 1.2:β = 0.985, a very small difference δ and bias (δ%) in the Bland–Altman method with no linearity between the difference and the average (*ρ*^2^ = 0.027), a weakly (CV, 4%) varying Oldham–Eksborg ratio of 1.04, and an AUC value very close to 0.5, indicating complete agreement between the methods. These results suggest that *r*^2^ alone is not a useful measure of agreement between two methods.

### 2.6. Measurement of Homoarginine in Mouse Plasma by GC-MS/MS and LC-MS/MS

hArg was measured in the plasma of mice by GC-MS/MS [86] and LC-MS/MS [71]. The hArg concentrations were measured as 245 ± 105 nM (CV, 43%) by the GC-MS/MS and 270 ± 204 nM (CV, 76%) by the LC-MS/MS. These values did not differ from each other (*p* = 0.190; two-tailed Wilcoxon test). The results of the methods-comparison are shown in Figure 5.

Linear regression analysis between the hArg concentrations measured by the LC-MS/MS (method 1) and those measured by the GC-MS/MS (method 2) resulted in a regression equation with a *y*-axis intercept value α = −186, a slope value β = 1.86, and a correlation coefficient (*r*^2^ = 0.9175). These observations suggest a good correlation between LC-MS/MS and GC-MS/MS, yet with the LC-MS/MS providing higher values for hArg in mouse plasma than the GC-MS/MS (Figure 5A).

The Bland–Altman approach revealed a moderate difference between the two methods (−25 ± 108 nM) according to a percentage difference of 13.6 ± 25% (bias) (Figure 5B1). The percentage difference (*y*) correlated weakly (*ρ*^2^ = 0.5421, *p* < 0.0001) with the average concentration (*x*): *y* = 80 − 0.26 × *x*. The difference correlated more strongly (*ρ*^2^ = 0.8601, *p* < 0.0001) with the average concentration of hArg: *y* = 80 − 0.258 × *x* (Figure 5B2). These findings argue for a moderate agreement between the GC-MS/MS and LC-MS/MS methods regarding hArg measurement in mouse plasma samples.

The approach according to Oldham and Eksborg resulted in the ratio Λ_OE_ = 0.978 ± 0.439 (CV, 45%) which is close to unity, but is considerably variable (Figure 5C).

The ROC approach resulted in an AUC value of 0.508 ± 0.021 and a *p* value of 0.8688, suggesting a good agreement between the two methods.

In summary, the Bland–Altman and the Oldham–Eksborg approaches indicate considerable disagreement between the GC-MS/MS and LC-MS/MS methods for hArg measurement in mouse plasma. Disagreement is especially visible at hArg concentrations lower than 200 nM (Figure 5), presumably because of the lower sensitivity of the LC-MS/MS method in terms of a higher limit of quantitation (LOQ).

The LC-MS/MS method for hArg was compared with an ELISA method for this amino acid in human plasma [71]. The linear regression analysis of the hArg concentrations measured by ELISA (y) correlated with those measured by LC-MS/MS: *y* = 0.04 + 0.76 × *x*, *r*^2^ = 0.78 [71]. Thus, LC-MS/MS yielded consistently higher hArg values than ELISA (*p* < 0.001). Analysis by the Bland–Altman approach resulted in a considerable difference of 0.50 ± 0.39 µM hArg between ELISA and LC-MS/MS. The data indicate a considerable disagreement between LC-MS/MS and ELISA for hArg measurement in human plasma.

### 2.7. Measurement of F_2_-Isoprostanes in Biological Samples

Free-radical-induced peroxidation of arachidonic acid and other polyunsaturated fatty acids esterified to lipids and subsequent hydrolysis generates prostaglandin-like compounds including isoprostanes and neuroprostanes. These compounds are accessible for quantitative determination in tissue, plasma and urine. F_2_-Isoprostanes have emerged as markers of lipid peroxidation in vivo in humans. Among them, 8-*iso*-prostaglandin (PG) F_2α_, also known as 8-*iso*-PGF_2α_, 8-*epi*-PGF_2α_, 15-F_2t_-IsoP, iPF_2α_-III, and its major urinary metabolites, i.e., 2,3-dinor-4,5-dihydro-8-*iso*-PGF_2α_ and 2,3-dinor-8-*iso*-PGF_2α_, were the subjects of extensive investigation [72,73,74,79,88,89,90,91]. Analytical approaches for F_2_-isoprotanes include chromatographic techniques such as thin-layer chromatography (TLC), high-performance liquid chromatography (HPLC), and GC, particularly in combination with MS. F_2_-Isoprostanes are extracted from biological samples by solid-phase extraction (SPE) or immunoaffinity column chromatography (IAC). HPLC and TLC are used for the isolation of particular F_2_-isoprostanes.

#### 2.7.1. Comparison between EIA and GC-MS

Figure 1 of the article by Devaraj et al. [73] shows the relationship for “F2Isoprostanes” in urine as measured by the authors’ group by using a commercially available EIA (i.e., method 1) and as measured by the group of Roberts by using a GC-MS method (i.e., method 2) after Morrow and Roberts [72].

The F_2_-isoprostanes concentration (mean ± SD) was measured as being 2.183 ± 1.623 ng/mg (CV, 74%) by the GC-MS (method 2) and 2.037 ± 1.135 ng/mg (CV, 56%) by EIA (method 1). The values did not differ between the methods (*p* = 0.612; two-tailed Wilcoxon test). The results of the methods-comparison are shown in Figure 6.

The linear regression analysis between the F_2_-isoprostanes concentrations measured in urine by EIA (method 1) and those measured by GC-MS (method 2) resulted in a regression equation with a *y*-axis intercept value α = 0.814, a slope value β = 0.560, and a correlation coefficient (*r*^2^ = 0.6422). These observations suggest a weak correlation between EIA and GC-MS (Figure 6A). On the basis of the data of Figure 6A, and on the assumption that the GC-MS method is the reference method, one may, at first glance, conclude that the EIA method is valid for the intended analyte. However, the calculated correlation coefficient *r*^2^ value of 0.64 across the whole concentration range is rather low. Nevertheless, such a value is frequently considered to be high enough throughout the literature.

The *y*-axis intercept α and the slope β values of the regression equation are often disregarded when methods are compared. The *y*-axis intercept value α of 0.81 of the regression equation in the whole concentration range of this example (Figure 6A) is clearly far from the zero which would indicate complete agreement. Moreover, this value may suggest that the LOQ value of the EIA method is about 0.8 ng/mg creatinine for the analyte in urine, with the lower values being most likely highly overestimated. The slope value β of 0.56 (for the whole range) suggests that the concentrations measured by this EIA method are statistically half of those measured by the GC-MS method. This finding seems to be supported by data in the literature [91] which suggest that the EIA method detects only one F2-isoprostane out of 64 potential isomers [89], i.e., 15(*S*)-8-*iso*-PGF_2α_, while the reported physicochemical methods, GC-MS [89] and GC-MS/MS [91], that do not use specific immunoaffinity column chromatography (IAC) extraction for 15(*S*)-8-*iso*-PGF_2α_, may detect an unknown number of additional F_2_-isoprostanes. The contribution of those additional F_2_-isoprostanes is presumably of the same extent (of about 50%) as that of 15(*S*)-8-*iso*-PGF_2α_ alone [91]. However, in the, by far more relevant, lower concentration range—both for controls and diabetes patients investigated in the study by Devaraj et al. [73]—there is no correlation between the EIA method and the GC-MS method (Figure 6A), i.e., *r*^2^ = 0.16 for the 0–3 range (*n* = 53) and *r*^2^ = 0.006 for the 0–3 range (*n* = 39). Figure 6A clearly demonstrates that the linearity between methods 1 and 2 solely is the result of very few (about 10% of the total) high concentration points (for a discussion see Ref. [24]). Thus, the value of linear regression analysis is limited, and the sole use of correlation coefficients may be misleading and even pretend correlation, because deviations in the lower concentration range are difficult to detect [8]. This comparison suggests that the correlation coefficient *r*^2^ = 0.64 is obviously too low and the agreement is analytically insufficient [90].

The Bland–Altman approach revealed a moderate difference between t5e two methods (−0.147 ± 0.985 ng/mg) according to a percentage difference of −1.07 ± 47% (bias) (Figure 6B). The percentage difference (*y*) correlated very weakly (*ρ*^2^ = 0.079, *p* = 0.022) with the average concentration (*x*): *y* = −22 + 10 × *x*. The difference (*y*) correlated very weakly (*ρ*^2^ = 0.279, *p* < 0.0001) with the average concentration (*x*): *y* = −0.68 + 0.392 × *x*. These findings argue for a weak agreement between the EIA and GC-MS methods with respect to F_2_-isoprostanes measurement in human urine samples.

The Bland–Altman approach (Figure 6B), and a deeper examination, reveal considerable disagreement between the EIA method and the reference GC-MS method [72]. The disagreement applies to the vast majority of those concentration points in the relevant concentration range for these substances [89], i.e., for the lower concentration range (see also Refs. [74,90]). Interestingly, the mean difference between the two methods is 0.15 (see Formula (2)), which is considerably low and close to zero. However, the standard deviation of the mean difference is 0.98, which is high and within the range of measured concentrations.

The approach according to Oldham and Eksborg resulted in the ratio Λ_OE_ = 1.159 ± 0.691 (CV, 60%) which is not very far from unity but is very variable (Figure 6C). This indicates a rather poor agreement between the EIA and the GC-MS methods, notably in the lower and more relevant concentration range (Figure 6C).

The ROC approach resulted in an AUC value of 0.510 ± 0.051 and a *p* value of 0.8413, suggesting agreement between the two methods.

#### 2.7.2. Comparison between ELISA and LC-MS/MS

Figure 5 of the article by Yan et al. [74] shows the linear regression between “iPF_2α_-III by ELISA (pg/mL)” and “iPF_2α_-III by LC-MS/MS (pg/mL)” in urine as measured by the authors themselves by using a commercially available ELISA (i.e., method 1) and as measured by the same group by using an LC-MS/MS method (i.e., method 2). It should be noted that iPF_2α_-III and 8-*iso*-PGF_2α_ are abbreviations for the same F_2_-isoprostane [88]. The largest part of the originally reported data of the study by Yan et al. [74], i.e., 67 out of 86 (78% of total) could be re-evaluated by the author of the present article and they are presented and discussed below.

The F_2_-isoprostane iPF_2α_-III concentration (mean ± SD) was measured to be 934 ± 506 pg/mL (CV, 54%) by ELISA (method 2) and 417 ± 241 pg/mL (CV, 58%) by LC-MS/MS (method 1). The values differed between the methods (*p* < 0.0001; two-tailed Wilcoxon test). Other results of the methods-comparison are shown in Figure 7.

Linear regression analysis between the iPF_2α_-III concentrations measured in urine by ELISA (method 1) and those measured by LC-MS/MS (method 2) resulted in the regression equation with a *y*-axis intercept value α = 176, a slope value β = 1.82, and a correlation coefficient (*r*^2^ = 0.7518). These observations suggest a weak correlation between the ELISA and LC-MS/MS methods (Figure 7A).

Yan et al. reported in their article that ELISA and LC-MS/MS provided very similar results [74]. However, the ELISA method provides on average about two times higher values than the LC-MS/MS method. It is worth mentioning, that the theoretical value of the slope of the regression line should be about 0.5 [79]. This is because ELISA detects most likely only one F_2_-isoprostane (*S*-form), while LC-MS and LC-MS/MS detect at least two F_2_-isoprostanes (*R*- and *S* forms). Thus, actually the ELISA method provides values, which are on average about 4 times higher than those measured by the LC-MS/MS method.

The Bland–Altman approach revealed a considerably difference between the two methods (−517 ± 320 pg/mL) according to a percentage difference of 75 ± 29% (bias) (Figure 7B). The percentage difference did not correlate with the average concentration. However, the mean difference correlated with the average (*y* = −9.2 − 0.752 × *x*, *ρ*^2^ = 0.7253, *p* < 0.0001), indicating a considerable proportional error of the ELISA method. These findings argue for a weak agreement between the ELISA and LC-MS methods with respect to F_2_-isoprostanes measurement in human urine samples.

The approach according to Oldham and Eksborg resulted in the ratio Λ_OE_ = 2.412 ± 0.920 (CV, 38%) which is far from unity (Figure 7C) and supports a disagreement between ELISA and LC-MS/MS, notably in the lower concentration range (Figure 7C). This example supports the critique by Altman and Bland on the use of the correlation coefficient for evaluating method-agreement of clinical measurement [10], even when the correlation coefficient value is fairly high (*r*^2^ = 0.75) as in the present example (Figure 7A).

The ROC approach yielded an AUC value of 0.845 ± 0.033 and a *p* value < 0.0001, suggesting a very low extent of agreement between the two methods.

#### 2.7.3. Comparison between GC-MS and LC-MS

Sircar and Subbaiah [90] have compared their LC-MS method for 15(*S*)-8-*iso*-PGF_2α_ (due to the use of IAC extraction) with the GC-MS method of Morrow and Roberts [72], i.e., which measures 15(*S*)-8-*iso*-PGF_2α_ and additional F_2_-isoprostanes in urine (Figure 8).

The F_2_-isoprostane 15(*S*)-8-*iso*-PGF_2α_ concentration (mean ± SD) was measured to be 0.825 ± 0.349 ng/mL (CV, 42%) by LC-MS (method 1) and 3.605 ± 1.362 ng/mL (CV, 38%) by GC-MS (method 2). The values differed between the methods (*p* < 0.0001; two-tailed paired *t* test). The results of the methods-comparison are shown in Figure 8.

Linear regression analysis between the iPF_2α_-III concentrations measured in urine by GC-MS (method 2) and those measured by LC-MS (method 1) resulted in the regression equation with a high *y*-axis intercept value α = 0.996, a high slope value β = 3.62, and a low correlation coefficient (*r*^2^ = 0.6555) (Figure 8A). These observations suggest a weak correlation between GC-MS and LC-MS.

The Bland–Altman approach revealed a moderate difference between the two methods (−0.147 ± 0.985 ng/mg; CV, 335%) according to a percentage difference of 125 ± 15% (bias) (Figure 8B). The percentage difference did not correlate with the average concentration (*ρ*^2^ = 0.028, *p* = 0.643). These findings argue for a weak agreement between the GC-MS and LC-MS methods. The difference correlated with the average concentration (*ρ*^2^ = 0.906, *p* < 0.0001). These findings argue for a weak agreement between the GC-MS and LC-MS methods with respect to the iPF_2α_-III measurement in human urine samples.

The approach according to Oldham and Eksborg resulted in the very high ratio Λ_OE_ = 4.513 ± 1.143 (CV, 25%) which is far from the unity (Figure 8C). However, such a high ratio would be expectable because the GC-MS measures several F_2_-isoprostanes in addition to 15(*S*)-8-*iso*-PGF_2α_ [72], while in the LC-MS method measures only 15(*S*)-8-*iso*-PGF_2α_ due to the use of a specific IAC extraction. The informative value of this comparison is considered low because of the small number of urine samples (*n* = 10).

The ROC approach resulted in an AUC value of 0.990 ± 0.016 and a *p* value of 0.0002, suggesting no agreement between the two methods.

### 2.8. An Example for Clinical Measurement-Systolic Blood Pressure [75,76]

Figure 9 shows the results from the application of the three method-comparison approaches to an example for a typical clinical measurement, i.e., for measuring systolic blood pressure (SBP) in 25 patients with essential hypertension, which was reported elsewhere [75,76]. In order to be able to evaluate the data in the same manner as in the examples discussed above, one of the methods used to measure blood pressure was chosen arbitrarily as the reference method (i.e., method 2) by the author of the present article.

The SBP values were measured to be 167 ± 25 mmHg (CV, 15%) by method 2 and 178 ± 29 mmHg (CV, 16%) by method 1, and were found to differ significantly (*p* < 0.0001; two-tailed paired *t* test), albeit by a relatively low extent of 6%.

Linear regression analysis between the two methods resulted in the regression equation with a *y*-axis intercept value α = −7, a slope value β = 1.11, and a correlation coefficient (*r*^2^ = 0.9113) (Figure 9A). These observations suggest a good correlation between the two methods of blood pressure measuring, with method 2 providing on average 1.1 times higher SBP values.

The Bland–Altman approach revealed a moderate difference between the two methods according to a percentage difference of −6 ± 4.6% (bias) (Figure 9B).

The approach according to Oldham and Eksborg resulted in the ratio Λ_OE_ = 0.943 ± 0.044 (CV, 5%) which is close to the unity and little variable (Figure 9C).

All three approaches indicate a considerable agreement between the compared methods of SBP measurement (Figure 9). The ROC approach resulted in an AUC value of 0.609 ± 0.080 and a *p* value of 0.1870, also suggesting agreement between the two methods.

### 2.9. A Second Example for Clinical Measurement-Peak Expiratory Flow Rate [10]

Figure 10 shows the results from the application of three method-comparison approaches to a clinical measurement, i.e., for measuring peak expiratory flow rate (PEFR) by two methods in 17 subjects, originally reported by Bland and Altman [10].

The PEFR values were measured to be 450 ± 116 mL/min (CV, 26%) by method 2 and 453 ± 113 mL/min (CV, 25%) by method 1, and differed significantly (*p* < 0.0001; two-tailed paired *t* test), albeit by a relatively low extent of 0.7%.

Linear regression analysis between the two methods resulted in the regression equation with a *y*-axis intercept value α = 39, a slope value β = 0.917, and a correlation coefficient (*r*^2^ = 0.8898) (Figure 10A). These observations suggest a good correlation between the two methods of PEFR measuring, with method 2 providing on average 0.92 times lower PEFR values.

The Bland–Altman approach revealed a small difference between the two methods (−2.1 ± 39 mL/min), yet with a considerable variability according to a percentage difference of −1 ± 12% (bias) (Figure 10B). The difference did not correlate with the average PEFR.

The approach according to Oldham and Eksborg resulted in the ratio Λ_OE_ = 0.995 ± 0.114 (CV, 5%) which is very close to the unity and little variable (Figure 10C).

The ROC approach resulted in an AUC value of 0.509 ± 0.1015 and a *p* value of 0.9314, also suggesting good agreement between the two methods of PEFR measurement.

## 3. Discussion

In the present work, published data from studies reporting on method-comparison were re-analyzed and re-evaluated by three method-comparison approaches, i.e., linear regression (LR) analysis and the Bland–Altman (BA) method, which are the most frequently used approaches, and the Oldham–Eksborg (OE) method, which is much less frequently used in comparison of analytical methods. The Oldham–Eksborg method is closely comparable with the Bland–Altman variant, in which the ratio of the results provided by two methods is plotted against their average. In the studies considered here, one of the applied methods was based on mass spectrometry, i.e., GC-MS, GC-MS/MS, LC-MS or LC-MS/MS. The analytes measured by the methods are all physiological low-molecular-mass substances, belong to different chemical classes and pathways, and require chemical derivatization for analysis by GC-MS-based methods (Scheme 1). Chosen biological matrices were human biological samples including plasma, serum and urine. The results of the present work are summarized in Table 1 and selected examples are illustrated in the Figure 1, Figure 2, Figure 3, Figure 4, Figure 5, Figure 6, Figure 7, Figure 8, Figure 9 and Figure 10.

GC-MS/MS and LC-MS/MS methods were among the methodologies used in these comparative studies. They are generally considered best useful as reference methods, i.e., the *Gold Standards*. The tandem mass spectrometry technique, when used in combination with chromatography, e.g., GC or LC (Schemes S1 and S2) in analytical chemistry, allows to generate accurate quantitative results, i.e., the concentrations of analytes in complex biological samples, even if the analytes are isomeric (Figure S2). It is considered that they are applied properly [48]. Analytical methods based on GC-MS/MS or LC-MS/MS are superior to those based on GC-MS or LC-MS, respectively, because the tandem mass spectrometry (MS/MS) is able to exclude (entirely) potentially interfering, mostly unknown analytes (Schemes S1 and S2). Of particular importance is the unique feature of MS to enable use of stable-isotope-labelled analogs of the analytes as internal standards in quantitative analyses. In contrast to the high specificity of MS-based methods, analytical methods that utilize much less specific physicochemical properties of analytes, such as light absorbance or fluorescence detection, even in combination with GC or LC, are generally considered less accurate because of the susceptibility to interferences and a lack of analytical sensitivity, i.e., too-high limits of detection. On the basis of these facts, MS/MS methods may reasonably be considered superior to MS methods on the one hand, and MS methods superior to non-MS methods, such as GC coupled to flame ionization detection (FID) or electrochemical detection (ECD), and LC coupled to UV/vis absorbance or fluorescence detection, on the other hand. Eventually, non-MS methods may be considered superior to batch assays which are free of any chromatographic or immunologic separation. Well-documented examples for batch assays include the analysis of nitrite based on the Griess reaction [92] and of malondialdehyde (MDA) based on the use of thiobarbituric acid (TBA) [93,94]. In a comparison of analytical methods, those based on GC-MS/MS and LC-MS/MS can serve as reference methods not only for those based on GC-MS and LC-MS, but also for non-MS-based analytical methods including immunological methods. Although this thought is widely spread, few scientists practise this principle in the field of bioanalysis.

In the present work, the Bland–Altman approach, the Oldham–Eksborg approach and the standard linear regression analysis were applied to compare published analytical methods for the measurement in biological samples of a series of structurally different physiological substances. Examples were taken from the author’s group and other groups who reported data from the use of two different analytical methods. Evaluations of comparability and agreement can be performed by using characteristic, preferably dimensionless, parameters of the abovementioned approaches. They include slope (β) and coefficients of correlation (*r*^2^), the Oldham–Eksborg ratio (Λ_OE_), the percentage difference, i.e., the bias (δ (%)), as well as the coefficient of correlation (*ρ*^2^) obtained from linear regression analysis of the difference δ versus the average μ in the Bland–Altman approach. The relation between difference and mean in the Bland–Altman approach was addressed by Bland and Altman, who found that in some cases the difference δ may be proportional to the mean μ [10], yet it was not further considered. This issue was addressed by Ludbrook [25]. Sporadically, such as in ophthalmology and vision science, exact parametric confidence intervals for the Bland–Altman approach were proposed and reviewed [28,95]. These approaches do not consider additional methods such as those investigated in the present work. In the present study, AUC data obtained from ROC analyses were also considered, an approach that is rarely used in comparisons of analytical methods.

Statistically significant differences between the τ_1_ and τ_2_ values may not indicate disagreement between the methods. Values of β, *r*^2^ and Λ_OE_ of the order of 1.00, δ(%) and *ρ*^2^ values close to 0.00, and ROC-AUC (AUC) values close to 0.5 would indicate perfect agreement between the two methods compared. The present study indicates that perfect agreement is an exception rather than a rule. By contrast, β, *r*^2^ and Λ_OE_ values different from 1.00, δ (%) and *ρ*^2^ values different from 0.00, and AUC values different from 0.5 would also not decisively indicate disagreeing methods. Rather, like in statistical analyses where statistical significance is defined arbitrarily, for instance *p* < 0.05, assessing the extent of agreement or disagreement of two methods demands definition of ranges rather than discrete values for *p*, β, *r*^2^, Λ_OE,_ δ (%) and possibly for AUC as well. As the definition of values and ranges is, per se, arbitrary, agreement or disagreement between two methods would also be arbitrary and relative. This resembles in many aspects the validation of analytical methods for which quantitative criteria were achieved by consensus and are widely used in the field of analytical chemistry for various types of analytical chemistry. These criteria include the precision in terms of the relative standard deviation (RSD) or the coefficient of variation (CV), the accuracy of the method in terms of recovery (%) for analytes added to biological samples at relevant concentrations, their limits of detection (LOD) and quantitation (LOQ), usually on the basis of the signal-to-noise (*S/N*) ratio. Such criteria have not been declared for the agreement or disagreement of methods, irrespective of the method-comparison approach. This is particularly the case with the Bland–Altman approach, which is mostly “degraded” to a simple plot.

On the basis of the results reported in the present work, each of the three currently available method-comparison approaches alone may be useful for comparing analytical methods, and for finding out which of the two compared methods is able to provide better, specifically more accurate, results. Yet, without a definition of reference methods and without a definition of quantitative criteria for the extent of agreement between two methods, no objective assessment is possible. A definition of acceptance criteria for main characteristic parameters for each method-comparison is required.

Yet, a reliable solution to this problem is likely to require the definition of a composite of all single parameters: *p*, β, *r*^2^, δ(%), *ρ*^2^, Λ_OE_, AUC. Such a composite may provide maximum information about agreement or disagreement between the two analytical methods being compared. A way to overcome this dilemma could be to accept fully validated and published MS/MS-based methods as the reference methods. This assumption is reasonable and justified because of the inherent accuracy of the MS/MS-technique, provided it is performed correctly and errors, such as contamination, artificial formation, or the degradation of analytes during sampling, sample storage, derivatization, and analysis, are eliminated [47,48]. Strictly speaking, comparisons of two methods require the use of validated protocols for each method and the performance of comparison studies in parallel under optimum conditions for each method [96,97], and should also include the use of standardized reference compounds for an analyte and its stable-isotope-labelled analog in GC-MS/MS and LC-MS/MS methods (see Figure 3) [68,69].

Special emphasis and consideration should be given when comparing chemical and immunological methods or immunological methods such as immunoaffinity chromatography (IAC) that are used for the isolation of certain analytes from biological samples prior to chemical analysis. Without a consideration of such aspects, considerable disagreement between two methods is expected to be observed, as is shown in the present work for F_2_-isoprostanes (Figure 6 and Figure 7) [72,73,74,79,88,89,90,91].

Most frequently, the Bland–Altman approach plots the absolute difference of two methods versus the average, as originally proposed by Bland and Altman [10]. Plotting the percentage difference against the average of two methods is also widely used. In the present work, two examples were presented which indicate that only one of the two Bland–Altman plots may reveal additional information about the agreement/disagreement between the methods which has not been reported by Bland and Altman. Observation of a linearity between the difference of two methods and their average is often interpreted as a systematic error and is even used to identify systematic errors [70,97,98]. In contrast, the lack of linearity between the percentage difference of two methods and their average may erroneously exclude the presence of a systematic error. It is therefore advisable to test this kind of linearity.

The analysis of the results observed in the present work (Table 1), revealed that the *p* value from the Wilcoxon test correlated inversely with the AUC value from the ROC analysis: *r* = −0.662, *p* = 0.042. The *r*^2^ value from the linear regression analysis correlated inversely with the δ(%) value of the Bland–Altman assay: *r* = −0.699, *p* = 0.029, and with the CV value from the Oldham–Eksborg test: *r* = −0.780, *p* = 0.010. The δ(%) value from the Bland–Altman test correlated directly with the correlation of coefficient from the linear regression analysis of the Bland–Altman difference vs. the average concentration *ρ*^2^: *r* = 0.729, *p* = 0.021, as well as with CV value from the Oldham–Eksborg test: *r* = 0.669, *p* = 0.039. Such correlations may suggest that many parameters rather than a single parameter of the three methods-comparison approaches may be useful in assessing agreement and to determine its extent.

Figure 11 shows the results separately for *p* (Wilcoxon or paired *t* test*)*, β, *r*^2^, *ρ*^2^, Λ, AUC, δ(%) and CV_OE_ taken from 10 examples listed in Table 1.

**Figure 12:**
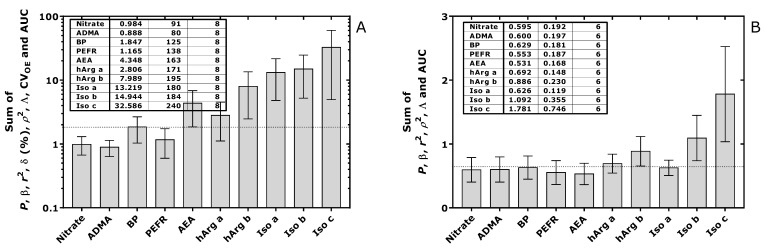
(**A**) Sum of values for selected statistical parameters taken from Table 1 obtained from the Wilcoxon or paired *t* test (*p*), the linear regression analysis (β and *r*^2^), the coefficient of correlation *ρ*^2^ from the linear regression analysis of the Bland–Altman difference δ vs. the average concentration of the analyte, the Oldham–Eksborg ratio Λ, the AUC value from the ROC analysis, the percentage difference of the Bland–Altman test δ(%), and the coefficient of variation of the Oldham–Eksborg ratio CV_OE_. (**B**) Like in (**A**), yet without δ(%) and CV_OE_ in order to exclude high values. Data from 10 examples were used. Note the decadic logarithmic scale on the *y* axis in panel (**B**). Data are shown as median with 95% confidence interval. Iso, F_2_ isoprostane. Insets indicate the tested parameters and their values. BP, systolic blood pressure; Iso, F_2_-isoprostanes. See Table 1.

Figure 12 shows the results for the sum of the statistical parameters for each analyte in these examples. The analytes included (*n* = 8) were nitrate, ADMA, AEA, hArg, F_2_-isoprostanes (Iso), SPB and PEFR. The numerically highest values were observed for δ (%) and CV_OE_ with respect to the statistical parameters, and for hArg (case hArg b) and the F_2_-isoprostanes and CV_OE_ with respect to the analytes.

## 4. Proposal of Criteria for Method Agreement

**Figure 13:**
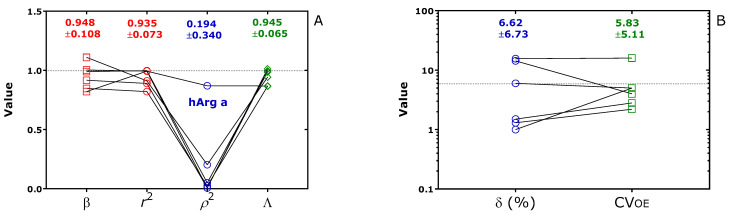
Presentation of values for selected statistical parameters obtained from the linear regression analysis (β and *r*^2^), the coefficient of correlation *ρ*^2^ from the linear regression analysis of the Bland–Altman difference δ vs. the average concentration of the analyte, and the Oldham–Eksborg ratio in (**A**); and for δ (%) and the CV_OE_ in (**B**). Lines combine symbols of the same example. Insets indicate the mean ± standard deviation of the statistical parameters from six examples (i.e., Nitrate, ADMA, AEA, hArg, BP, PEFR). Note the decadic logarithmic scale on the *y* axis in panel (**B**). See Figure 11.

The results of the present study suggest that reliable comparison of two analytical methods is best performed by using a combination of different statistical methods. Statistical difference between the concentrations of an analyte measured in a biological sample is not useful in assessing agreement. We can dispense with the use of the paired *t* test or Wilcoxon test. Linear regression analysis is useful, but we need not only the coefficient of correlation (*r* or *r*^2^), but also the slope β of the regression line. The closer *r* and β to a value of 1.0, the higher the extent of agreement. However, linear regression does not reveal potentially important differences between the methods. This can be observed by the Bland–Altman approach, when the difference δ_AB_ is proportional to the mean μ_AB_. The coefficient of correlation *ρ*^2^ in the Bland–Altman approach is a useful parameter in assessing agreement. This is clearly visible in Figure 13A, notably in the measurement of hArg in plasma by GC-MS and GC-MS/MS. The Oldham–Eksborg approach provides values of the ratio Λ which correlate with β and *r*^2^. Thus, the closer Λ to the value of 1.0, the higher the extent of the agreement. The bias, i.e., the percentage difference between the methods δ(%) in the Bland–Altman approach correlates with the coefficient of variation CV_OE_ of the Oldham–Eksborg ratio Λ (Figure 13B).

The absolute value of the difference δ is less informative. It can therefore be concluded that β, *r*^2^, *ρ*^2^, Λ_OE_, δ (%) and CV_OE_ are useful in evaluating method agreements and in determining the extent of agreement between two analytical methods. Figure 13 suggests that good agreement between two methods exists when β, *r*^2^, Λ_OE_ do not differ from 1.00 by 7 to 11%, and δ (%) and CV_OE_ are below 12%.

In an analogy to currently available guidelines for chemical analytical methods with respect to method validation [52,53,54,55,56,57,99], acceptance criteria for agreement could be defined as ±15% from 1.00 for β, *r*^2^, Λ_OE_ and ±15% for δ (%).

## Figures and Tables

**Table 1:** Summary of the results from the re-evaluation of published studies by using linear regression analysis, the Bland-Altman and the Oldham-Eksborg method, and the ROC approach between two methods.

	Mean		*n*	*p* Value	Linear Regression			Bland-Altman				Oldham-Eksborg		ROC		Method 1/Method 2	Refs.
No	τ_2µ_	τ_1µ_	*n*	*p*	α	β	*r* ^2^	δ	CV (%)	δ (%)	*ρ*^2^ (δ; δ%)	Λ	CV (%)	AUC ± SE	*p*		
**(1) Nitrate in urine (a, b) and plasma (c) (µM)**																	
a	1059	1048	20	0.0136	1.24	0.99	0.998	−12 ± 50	1.1 ± 4.7	−1.5 ± 2.7	0.05; 0.02	0.986 ± 0.027	2.8	0.531 ± 0.093	0.735	GC-MS/GC-MS/MS	[58]
b	885	907	240	0.554	−121	1.16	0.973	30 ± 183	3.4 ± 21	n.d.		0.983 ± 0.231	23	n.d.		HPLC/GC-MS	[59,60]
c	41	36	40	0.0002	−21	1.37	0.896	−5.5 ± 8.7	13 ± 21	n.d.		0.822 ± 0.253	31	n.d.		Griess/GC-MS	[61]
**(2) Asymmetric dimethylarginine (ADMA) in urine (a) and plasma or serum (aa, b–g) (µM or nM)**																	
aa	1351	1334	18	0.034	12.7	1.003	0.994	17 ± 1.2	29 ± 180	1.3	0.007; 0.001	1.013 ± 0.0219	2.2	0.549 ± 0.097	0.613	Serum/Plasma	[62]
a	14.9	13.2	10	0.008	0.22	1.100	0.9997	1.16 ± 1.5	12 ± 11	n.d.		1.170 ± 0.097	8.3	n.d.		GC-MS/GC-MS/MS	[63]
b	654	640	10	0.072	2.37	1.020	0.9966	14 ± 21	2.2 ± 3.3	n.d.		1.02 ± 0.03	3.4	n.d.		GC-MS/GC-MS/MS	[63]
c			9		−0.9	0.999	0.991									ELISA/GC-MS	[64]
d			29		0.01	0.85	0.984									ELISA/LC-MS/MS	[64]
e	0.72	0.37	11		0.40	0.34	0.312									ELISA/LC-MS/MS	[65]
f			55													ELISA/HPLC	[66]
g	1.75	0.83	36	1.3 × 10^−18^	−0.19	0.86	0.944	0.9 ± 0.3	108 ± 36			2.09 ± 0.24				ELISA/HPLC	[67]
**(3) Anandamide (AEA) in plasma (nM)**																	
	0.844	0.729	305	<0.0001	0.013	0.848	0.821	0.116 ± 0.123	106	15.6 ± 16.5	0.024; 0.024	0.866 ± 0.136	16	0.626 ± 0.024	<0.0001	GC-MS/MS/LC-MS/MS	[68,69]
**(4) Homoarginine (hArg) in plasma (µM or nM)**																	
a	0.744	0.643	369	<0.0001	0.03	0.821	0.994	0.105 ± 0.070		14.3 ± 27	0.871; 0.174	0.868 ± 0.035	4	0.596 ± 0.021	<0.0001	GC-MS/GC-MS/MS	[70]
b	245	270	79	0.190	186	1.86	0.918	−25 ± 108		13.6 ± 56	0.860; 0.542	0.978 ± 0.439	45	0.508 ± 0.021	0.869	LC-MS/MS/GC-MS/MS	[70,71]
**(5) 15(S)-8-iso-PGF2α and other F2-isoprostanes in urine**																	
a	2.813	2.037	66	0.612	0.814	0.560	0.642	−0.147±0.985	42; 38	−1.07 ± 47	0.272; 0.079	1.159 ± 0.691	60	0.510 ± 0.051	0.841	EIA/GC-MS	[72,73]
b	934	417	67	<0.0001	176	1.82	0.752	−517 ± 320		−75 ± 29	0.725; 0.005	2.412 ± 0.920	38	0.845 ± 0.033	<0.0001	ELISA/LC-MS/MS	[74]
c	3.605	0.825	10	<0.0001	0.996	3.62	0.656	147 ± 985	335	125 ± 15	0.906; 0.028	4.513 ± 1.143	25	0.990 ± 0.016	0.0002	GC-MS/LC-MS	[74]
**(6) Systolic blood pressure (mmHg)**																	
	167	178	25	<0.0001	−7	1.11	0.911	10.7 ± 9.0	84	−6 ± 4.6	0.203; 0.095	0.943 ± 0.044	5	0.609 ± 0.080	0.187	Apparat. 1/Apparat. 2	[75,76]
**(7) Peak expiratory flow rate (mL/min)**																	
	450	453	17	<0.0001	39	0.917	0.890	2.1 ± 39	1830	−1 ± 12	0.007; 0.058	0.995 ± 0.114	5	0.509 ± 0.102	0.931	Instrum. 1/Instrum. 2	[10]
**Miscellaneous**																	
**(8) Nitrite (a) and nitrate (b) in bronchoalveolar liquid (µM)**																	
a	0.5	1.71	72	<0.0001	0.07	0.25	0.395	−1.2 ± 0.6	70 ± 35			0.28 ± 0.20	71			CL/GC-MS	[77]
b	6.0	9.5	72	<0.0001	−0.35	0.67	0.946	−3.1 ± 4.0	33 ± 42			0.64 ± 0.21	33			CL/GC-MS	[77]
**(9) 3-Nitrotyrosine in plasma (nM)**																	
	4.8	1.65	18	0.002	3.06	1.04	0.334	3.1 ± 3.7	188 ± 224			3.9 ± 3.7	95			GC-MS/GC-MS/MS	[78]
**(10) 2,3-dinor-5,6-dihydro-PGF2α in urine**																	
	543	502	14	0.434	12	1.06	0.832	40 ± 186	8 ± 37			1.12 ± 0.59	53			GC-MS/GC-MS/MS	[79]
**(11) Creatinine in urine (mM)**																	
a	5.15	5.21	24	0.466	−0.13	1.01	0.998	−0.06 ± 0.40	1.2 ± 7.7			0.97 ± 0.07	7.2			HPLC/GC-MS	[80]
b	4.04	5.20	24		0.29	0.72	0.996	−1.17 ± 1.77	23 ± 34			0.82 ± 0.08	9.8			Jaffé/GC-MS	[80]
**(12) Total plasma homocysteine (µM)**																	
	13.6	12.7	31	<0.001	**2.47**	**0.88**	**0.990**	0.91 ± 1.31	7.2 ± 10			1.13 ± 0.14	12.4			FPIA/GC-MS	[63]

N.A., not available. Abbreviations: CL, chemiluminescence; FPIA, fluorescence polarization immunoassay.

**Table 2:** Linear regression, Bland–Altman, Oldham–Eksborg and ROC analysis using simulated homoarginine concentrations. The original concentrations measured by DSQ (GC-MS, i.e., DSQ × 1.0) were multiplied by 1.2, 0.8 and 0.6 and compared with those measured by TSQ (GC-MS/MS).

DSQ-Fold	Linear Regression		Bland–Altman				Oldham–Eksborg	ROC
DSQ × 1.2	*y* = 0.04 + 0.9846*x*	*r*^2^ = 0.9943	δ = −0.0234 ± 0.0281	CV = 117%	*ρ*^2^ = 0.0271	δ (%) = −3.94 ± 3.98 CV = 100%	1.041 ± 0.041 CV = 4%	0.5268 *p* = 0.2081
DSQ × 1.0	*y* = 0.03 + 0.8205*x*	*r*^2^ = 0.9943	δ = 0.1046 ± 0.0698	CV = 67%	*ρ*^2^ = 0.8701	δ (%) = 14.3 ± 3.9 CV = 28%	0.868 ± 0.034 CV = 4%	0.5961 *p* < 0.0001
DSQ × 0.8	*y* = 0.02 + 0.6564*x*	*r*^2^ = 0.9943	δ = 0.2331 ± 0.1276	CV = 55%	*ρ*^2^ = 0.9699	δ (%) = 36.2 ± 3.8 CV = 11%	0.694 ± 0.028 CV = 4%	0.7285 *p* < 0.0001
DSQ × 0.6	*y* = 0.02 + 0.4923*x*	*r*^2^ = 0.9943	δ = 0.3617 ± 0.1871	CV = 51%	*ρ*^2^ = 0.9903	δ (%) = 63.2 ± 3.6 CV = 6%	0.520 ± 0.021 CV = 4%	0.8595 *p* < 0.0001

## Data Availability

The study did not report any data.

## Supplementary Materials

The following supporting information can be downloaded at: https://www.mdpi.com/article/10.3390/molecules28134905/s1. Figure S1. Number of yearly citations of the paper by (A) J.M. Bland and D.G. Altman [10] and by (B) M.M. Bradford [100] according to Scopus (Elsevier) from 1976 to 11 January 2023. Bland and Altman reported an approach on method comparison, which is widely known as the Bland–Altman plot [10]. Bradford reported in her paper a method for the measurement of protein concentration utilizing the principle of protein-dye binding [100]. The paper by M.M. Bradford is thematically not related to the present work but is suitable for a better understanding of the value of the paper by J.M. Bland and D.G. Altman in science. Scheme S1. Schematic of the principles of the mass spectrometry (MS) and tandem mass spectrometry (MS/MS) based on the quadrupole (Q) technology, exemplified for two structurally closely related analytes A and B which co-elute (same retention time, *t*_R_) and ionize to form two isobaric ions (same mass-to-charge, *m*/*z*, ratio). (Upper left) Analytes A and B cannot be discriminated by single-stage quadrupole (SSQ) MS spectrometers. (Upper right, lower left) In the collision chamber (i.e., the second quadrupole Q2) of triple-stage quadrupole (TSQ) MS/MS spectrometers, collision induced dissociation (CID) of the precursor ions A and B with argon atoms produces several common and two distinctly different products ions (indicated by dotted arrows). (Lower, right) The third quadrupole Q3 of TSQ MS spectrometers separates the different product ions (set in dotted circles) formed in Q2. Thus, unlike SSQ MS spectrometers, TSQ MS/MS spectrometers can discriminate between analytes that co-elute and ionize in the ion source to form isobaric ions (same *m/z*). CID in Q2 and subsequent second mass separation in Q3 in TSQ instruments and related MS/MS instruments guarantee unique specificity. This feature makes MS/MS-based analytical methods the most qualified candidates to serve as reference methods, as the Gold Standard, for numerous analytes. See also Figure S2 and Scheme S2. Scheme S2. Schematic of the most frequently used modes in quantitative analyses of a target analyte A by using its stable-isotope-labelled analogue serving as the internal standard on quadrupole instruments. (A) Selected-ion monitoring (SIM) by mass spectrometry (MS) and (B) Selected-reaction monitoring (SRM) by tandem mass spectrometry (MS/MS). For more, details see the text. Figure S2. GC-MS (A,B) and GC-MS/MS (C,D) spectra of the pentafluorobenzyl (PFB) esters of 9-nitro-oleic acid (9-NO_2_-OA) and 10-nitro-oleic acid (10-NO_2_-OA). Electron-capture negative-ion chemical ionization (ECNICI) of the PFB esters of 9-NO_2_-OA (A) and 10-NO_2_-OA (B) leads to almost identical mass spectra, with the most intense ion being [M–PFB]^−^ with *m*/*z* 326. In GC-MS/MS, the isobaric (*m*/*z* 326) parent (P) ions ([M–PFB]^−^, [P]^−^) are separated by Q1, subjected in Q2 to collision induced dissociation (CID), and the product ions formed in Q2 are separated by Q3. The product ion mass spectra of 9-NO_2_-OA (C) and 10-NO_2_-OA (D) are different. The product ions *m/z* 195 and *m/z* 197 are produced from 9-NO_2_-OA, but not from 10-NO_2_-OA. Thus, 9-NO_2_-OA and 10-NO_2_-OA can be discriminated by GC-MS/MS even if their PFB esters would co-elute. See also Scheme S1 and Ref. [101].

## Sample Availability

Not available.
