# Supplementary material for: Mass Spectrometry-Based Evaluation of the Bland–Altman Approach: Review, Discussion, and Proposal
Source: Molecules. 2023 Jun 21;28(13):4905. doi: 10.3390/molecules28134905 (PMC10343610; doi:10.3390/molecules28134905)
Supplement: Supplementary file 1 [file molecules-28-04905-s001.zip › molecules-2415610-supplementary.pdf]

Supplementary Materials

Mass Spectrometry-Based Evaluation of the Bland–Altman Approach: Review, Discussion, and Proposal

Dimitrios Tsikas

Institute of Toxicology, Core Unit Proteomics, Hannover Medical School, 30623 Hannover, Germany; tsikas.dimitros@mh-hannover.de

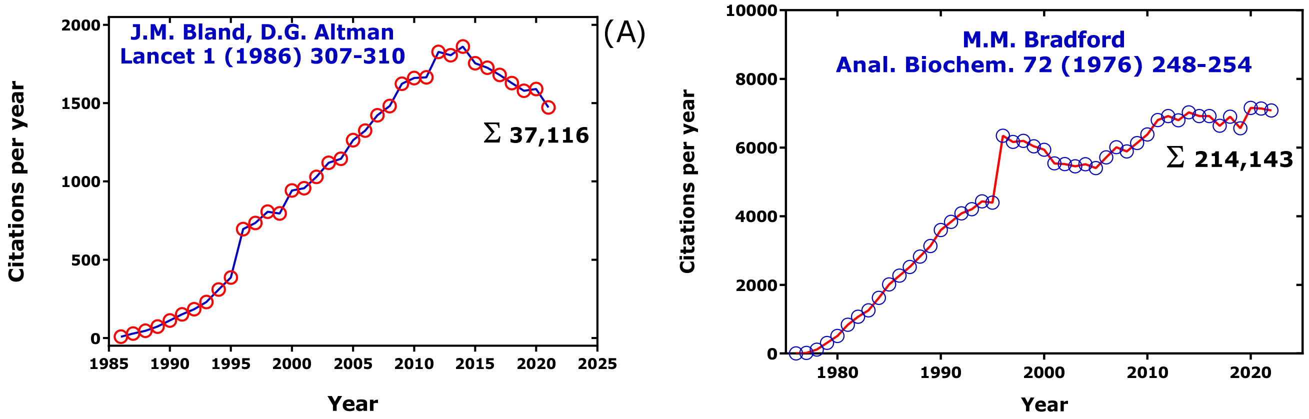

Figure S1. Number of yearly citations of the paper by (A) J.M. Bland and D.G. Altman [10] and by (B) M.M. Bradford [100] according to Scopus (Elsevier) from 1976 to 11 January 2023. Bland & Altman reported an approach on method comparison, which is widely known as the Bland-Altman plot [10]. Bradford reported in her paper a method for the measurement of protein concentration utilizing the principle of protein-dye binding [100]. The paper by M.M. Bradford is thematically not related to the present work, but is suitable for a better understanding of the value of the paper by J.M. Bland and D.G. Altman in science.

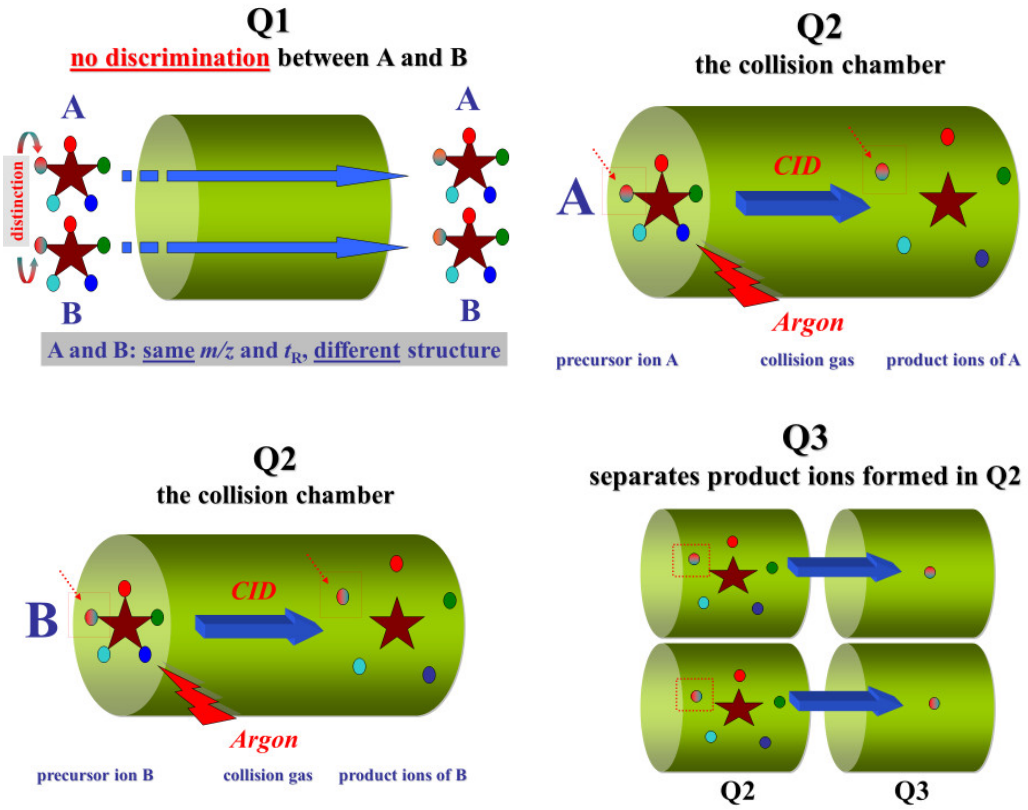

Scheme S1. Schematic of the principles of the mass spectrometry (MS) and tandem mass spectrometry (MS/MS) based on the quadrupole (Q) technology, exemplified for two structurally closely related analytes A and B which co-elute (same retention time, t_R) and ionize to form two isobaric ions (same mass-to-charge, *m*/*z*, ratio). (Upper left) Analytes A and B cannot be discriminated by single-stage quadrupole (SSQ) MS spectrometers. (Upper right, lower left) In the collision chamber (i.e., the second quadrupole Q2) of triple-stage quadrupole (TSQ) MS/MS spectrometers, collision induced dissociation (CID) of the precursor ions A and B with argon atoms produces several common and two distinctly different products ions (indicated by dotted arrows). (Lower, right) The third quadrupole Q3 of TSQ MS spectrometers separates the different product ions (set in dotted circles) formed in Q2. Thus, unlike SSQ MS spectrometers, TSQ MS/MS spectrometers can discriminate between analytes that co-elute and ionize in the ion-source to form isobaric ions (same *m*/*z*). CID in Q2 and subsequent second mass separation in Q3 in TSQ instruments and related MS/MS instruments guarantee unique specificity. This feature makes MS/MS-based analytical methods the most qualified candidates to serve as *Reference Methods*, as the *Gold Standard*, for numerous analytes. See also Fig. 2S and Scheme 2S.

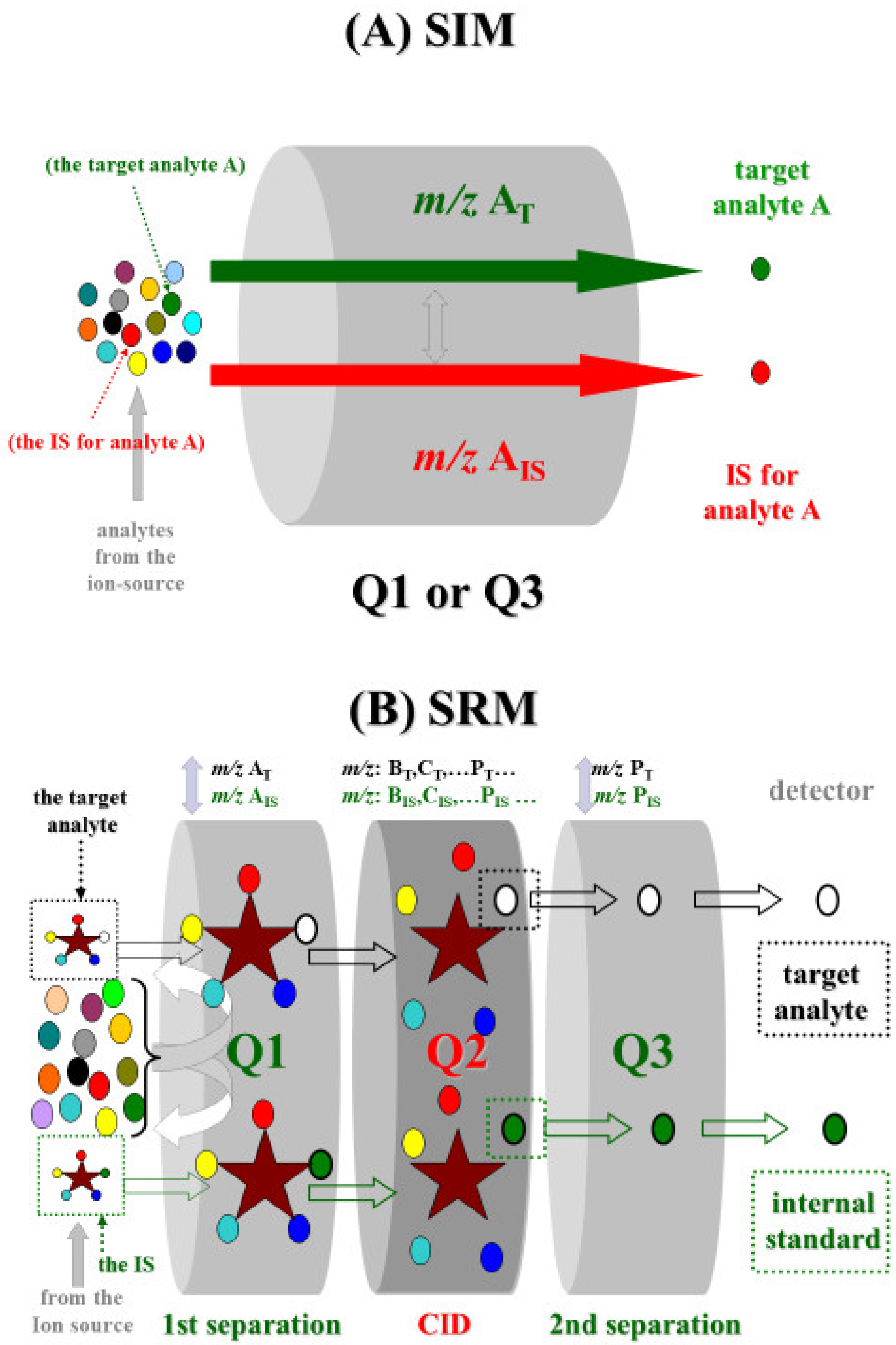

Scheme S2. Schematic of the most frequently used modes in quantitative analyses of a target analyte A by using its stable-isotope labelled analogue serving as the internal standard on quadrupole instruments. (**A**) Selected-ion monitoring (SIM) by mass spectrometry (MS) and (**B**) Selected-reaction monitoring (SRM) by tandem mass spectrometry (MS/MS). For more, details see the text. .

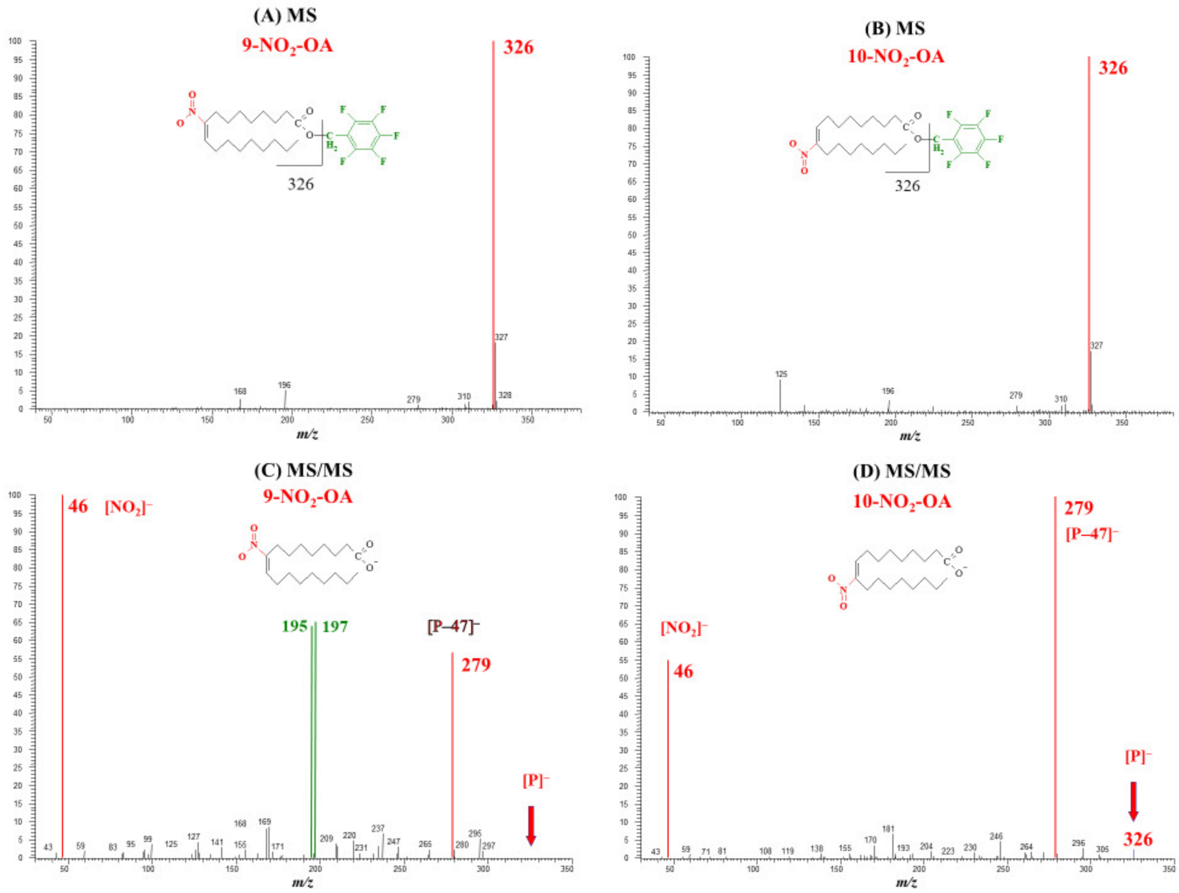

Figure S2. GC-MS (**A**,**B**) and GC-MS/MS (**C**,**D**) spectra of the pentafluorobenzyl (PFB) esters of 9-nitro-oleic acid (9-NO_2-OA) and 10-nitro-oleic acid (10-NO_2-OA). Electron-capture negative-ion chemical ionization (ECNICI) of the PFB esters of 9-NO_2-OA (A) and 10-NO_2-OA (B) leads to almost identical mass spectra, with the most intense ion being [M─PFB]⁻ with *m*/*z* 326. In GC-MS/MS, the isobaric (*m*/*z* 326) parent (P) ions ([M─PFB]⁻, [P]⁻) are separated by Q1, subjected in Q2 to collision induced dissociation (CID), and the product ions formed in Q2 are separated by Q3. The product ion mass spectra of 9-NO_2-OA (C) and 10-NO_2-OA (D) are different. The product ions *m*/*z* 195 and *m*/*z* 197 are produced from 9-NO_2-OA, but not from 10-NO_2-OA. Thus, 9-NO_2-OA and 10-NO_2-OA can be discriminated by GC-MS/MS even if their PFB esters would co-elute. See also Scheme 1S and Ref. [101].
